# Traffic flow detection method based on improved SSD algorithm for intelligent transportation system

**DOI:** 10.1371/journal.pone.0300214

**Published:** 2024-03-14

**Authors:** Guodong Su, Hao Shu

**Affiliations:** 1 School of Physics and Optoelectronics, Xiangtan University, Xiangtan, China; 2 Examination Center of Human Resources and Social Security Bureau of Changsha City, Changsha, China; Sunway University, MALAYSIA

## Abstract

With the development of the new generation communication system in China, the application of intelligent transportation system is more extensive, which brings higher demands for vehicle flow detection and monitoring. Traditional traffic flow detection modes often cannot meet the high statistical accuracy requirement and high-speed detection simultaneously. Therefore, an improved Inception module is integrated into the single shot multi box detector algorithm. An intelligent vehicle flow detection model is constructed based on the improved single shot multi box detector algorithm. According to the findings, the convergence speed of the improved algorithm was the fastest. When the test sample was the entire test set, the accuracy and precision values of the improved method were 93.6% and 96.0%, respectively, which were higher than all comparison target detection algorithms. The experimental results of traffic flow statistics showed that the model had the highest statistical accuracy, which converged during the training phase. During the testing phase, except for manual statistics, all methods had the lowest statistical accuracy on motorcycles. The average accuracy and precision of the designed model for various types of images were 96.9% and 96.8%, respectively. The calculation speed of this intelligent model was not significantly improved compared to the other two intelligent models, but it was significantly higher than manual monitoring methods. Two experimental data demonstrate that the intelligent vehicle flow detection model designed in this study has higher detection accuracy. The calculation speed has no significant difference compared with the traditional method, which is helpful to the traffic flow management in intelligent transportation system.

## 1. Introduction

With the acceleration of urbanization, transportation problems have gradually become an important factor restricting urban development. As an effective solution, Intelligent Transportation System (ITS) has attracted extensive attention from all walks of life. The ITS has realized the intellectualization of transportation system through advanced information technology, data communication, electronic control, vehicle engineering and other technologies. It has improved the operational efficiency and safety of the transportation system, optimized the travel environment, and provided strong technical support for the sustained and healthy development of urban transportation [1, 2]. Among numerous ITS applications, traffic flow detection is a key link. Vehicle flow is a considerable indicator to measure the operational status of road traffic, which is of great significance for traffic management departments to formulate reasonable traffic control strategies, optimize traffic resource allocation, and improve road utilization efficiency [3, 4]. At present, the technology roadmap mainly adopted in traffic flow detection includes sensor based detection method and video based detection method. The former has high accuracy. However, there are high installation and maintenance costs and significant environmental impacts [5]. The video detection method collects road traffic images through a camera. Through image processing and computer vision technology analysis, traffic flow data is obtained. This method has low installation and maintenance costs. However, in complex environments such as changes in lighting and occlusion, the accuracy is relatively low. In traffic environments, vehicle flow detection methods based on video data often struggle to detect small pixel vehicle targets. For vehicles with similar color and edge features to environmental pixels, the detection performance is also poor. This is mainly because the detection algorithm itself cannot adapt well to the feature extraction and recognition tasks of traffic flow detection. Therefore, it is necessary to establish a more intelligent traffic flow detection model.

To improve the detection accuracy of automated traffic flow detection models, a traffic flow detection method based on the fusion improved Single Shot MultiBox Detector (SSD) algorithm is proposed, providing technical support for the development of intelligent transportation systems. In traditional SSD algorithms, due to the limitations of convolutional module structure and background interference elements, the semantic information of the target vehicle in the generated feature map may be lacking. This results in insufficient detection capability of traditional SSD algorithms for small or distant vehicles, which is also a field with many gaps in image-based vehicle flow detection.

In response to the insufficient detection ability of traditional SSD algorithms for small volume and distant vehicles, this study has added an improved Inception structure to the SSD algorithm, hoping to enhance the algorithm’s recognition ability for small pixel features. This is also the main contribution of this study and an extension of the knowledge system in this field. This study can provide some higher-level intelligent monitoring methods for urban vehicle traffic safety monitoring and statistical applications.

This study consists of four parts. The first part introduces the development status of intelligent transportation and the role of traffic flow detection. The core content of the second part is to design a traffic flow detection model based on improved SSD algorithm and DeepSort, which is also the innovation of this study. The third part designs an experiment using the proposed model for detecting monitor video frame images. The findings are compared with some common models. The fourth part is to analyze the results obtained from the experiment.

## 2. Related works

The essence of traffic flow detection is image recognition. The video data is converted into video frame images. Then, image recognition is used to identify the cars in the video frame and count them, thereby deriving the traffic volume based on the cars appearing in the image within the specified time. Image recognition and object detection are popular fields in artificial intelligence and machine learning. A target detection method based on signal structure information was proposed by Gao C et al. [6], which utilizes a fully CNN to perform hierarchical learning of signal structure information. Research points out that traditional object detection methods often overlook some structural information introduced through matched filtering and coherent integration, which is crucial for improving detection performance. According to the findings, it can achieve better detection performance. The signal anomaly image recognition problem in passive phased array radar was discussed by Rad MK et al. [7]. An image recognition model based on the RestNet is designed. According to the findings, the proposed model not only improves the recognition accuracy of abnormal signals compared to the benchmark algorithm, but also has no significant increase in computational complexity. An improved target detection strategy based on Faster regional CNN was designed by Wang K et al. [8]. According to the findings, it has higher accuracy in different image object detection datasets. A universal and effective multipath attention mechanism (AM) was proposed by Zhang H et al. [9]. to explore the impact of visual attention on image recognition. This mechanism only generates a simple and unified computational block, which can be flexibly integrated into various CNNs with fewer parameters. It supports end-to-end training. The outcome indicates that the CNN with the AM has better performance than the original network.

In a recent study, a strong semantic weakly supervised edge detection algorithm was proposed by Zhao H et al. [10], which combines edge detection with image semantic segmentation. It can effectively detect important boundaries of spacecraft from the image, regardless of background interference and lighting intensity. The outcomes indicate that this algorithm can effectively extract the important boundaries of the spacecraft and filter out background information and noise. A point level vehicle detection method based on three-dimensional prior cyclic CNN for automatic driving was proposed by Tao C et al. [11]. in the study, which combines traditional Regional Proposal Network (RPN) and Mask-branch mechanism. It can minimize brightness error and improve the 3D object detection accuracy. RGB images are used to provide semantic information for spatial point clouds. The performance on the Kitti and nuScenes datasets proves the efficiency and effectiveness, but it can only be applied to autonomous driving scenarios. A generation data enhancement model was proposed by Hu WJ et al. [12]. in the study, which is essentially a depth convolution Generative adversarial network. Experiment shows that this model outperforms the baseline model in image generation and data enhancement, whether in the agricultural or medical fields. Compared with the baseline model, the recognition error rate is reduced by more than 4%. Simultaneously, this model is used for data augmentation, which significantly improves the image classification ability of designated industries. The average accuracy of classification results on agricultural and medical images is improved by 0.96% and 1.27% compared to the baseline model. Most of the above research focuses on static image recognition. Although some design ideas of image processing technologies are worth learning from, the algorithms and models designed in these studies cannot be directly applied to vehicle dynamic monitoring video image processing.

The task of traffic flow detection needs to maintain a balance between detection accuracy and running time. Therefore, the following previous studies can provide some ideas for this study. Dong Z et al. [13]. A neural morphology sensory processing system with a memristor circuit was designed by Dong Z et al. [13]. It can be used in smart homes to improve the detection efficiency and reduce detection costs. The test results show that the system effectively improves the interactive response and image detection speed of smart homes. A compact limit learning machine (ELM) structure composed of synaptic circuits, bias circuits, and activation function circuits based on spin electron memristors was proposed by Dong Z et al. [14]. The test results show that this structure can achieve better computational performance than traditional structures in applications. A circuit design based on the hierarchical attention network was proposed by Dong Z and Ji X et al. [15] for multimodal emotional computing. It can be used for mental health monitoring. Specifically, it prepares a cost-effective memristor. The test results show that the designed network and memristor can accurately monitor the current mental health status of users. The memristor technology is attractive due to its non volatile nature, high density, low power consumption, and compatibility with CMOS. Therefore, a flexible memristor model with resistive switching memory behavior is proposed by Ji X et al. [16]. in the study. The performance test results conducted on an electrochemical workstation indicate that this model contributes to construct higher operational quality neural network synaptic systems. A novel memristor model based on Ag/TiOx Nano-belt structure was designed by Ji X et al. [17]. It can reflect the initial stage, transition stage, and resistance switching state of physical memristors. Experimental results show that the fitting accuracy of this model is greater than 99.88%. A metal oxide based memristor with high stability, low power consumption, and good scalability was developed by Dong Z et al. [18]. It serves as a fundamental component of neural morphology computing systems. At the circuit level, the basic circuit units and necessary peripheral circuits are designed to achieve efficient vector matrix multiplication and different functions. A brain inspired hierarchical interactive memory computing (IMC) system was proposed by Ji X et al. [19]. The experimental results show that the system has high computational efficiency and good robustness. It is superior to existing advanced methods. An environmentally friendly memristor using two-dimensional materials was constructed by Ji X et al. [20]. The effectiveness and reliability are confirmed through performance testing. A sequencer block is proposed using a memristor circuit. The experimental results show that the proposed sequencer block has advantages in computational efficiency and classification accuracy compared to existing mainstream methods.

In terms of vehicle recognition, previous researchers have also conducted some research. The deep learning YOLOv3 algorithm was used by Rajput S K et al. [21] to achieve automatic vehicle recognition and classification. It is deployed in the toll management system of toll plazas. The experimental results show that the vehicle recognition performance of this algorithm is significantly better than the original algorithm [21]. However, the method designed in reference [21] performs poorly in high-speed and high traffic flow recognition tasks. Two vehicle recognition and license plate recognition models based on neural network algorithms were designed by Gonz á lez Ceeda J et al. Several datasets are created to validate the models. The verification results show that the model designed in the study has excellent recognition ability, which adapt to some extreme weather working environments [22]. However, similarly, the research results in reference [22] cannot be directly applied to traffic flow statistics work.

In summary, previous studies have conducted extensive research to improve the performance and computational speed of image recognition models. However, the detection model for the traffic flow statistics in intelligent transportation system is still less studied. Moreover, most of the methods obtained from these few studies lack universality.

## 3. The vehicle flow detection model design based on DeepSort and improved SSD algorithm

Accurate vehicle recognition and detection is the initial step of video image traffic flow detection algorithms. One of the research highlights in the intelligent transportation is vehicle detection, which is a key foundation for achieving technologies such as traffic flow detection and autonomous driving [23, 24]. However, in complex actual traffic environments, due to the vehicle distance under the camera’s perspective, the detection performance of small scale vehicles in the distance is usually poor, which is also a major challenge in the object detection [25]. To improve the detection accuracy of small-scale vehicles, an improved SSD small-scale vehicle detection algorithm is designed based on the original SSD algorithm. Firstly, the sparse connection concept in Inception is adopted in the study to optimize the convolutional layer structure for small-scale vehicle detection. This operation can enhance the feature extraction ability of SSD shallow convolutional networks to meet the traffic scene detection needs in various scales. Then, in the feature map (FM) processing stage, the Squeeze-and-Excitation Networks (SENet) channel AM is introduced to enhance the attention to channels with large amounts of information. This operation can improve the information expression ability of FMs to further upgrade the detection performance of small-scale vehicles.

### 3.1 Vehicle detection model based on improved SSD algorithm

Before designing an improved SSD algorithm, the limitations of traditional SSDs are discussed. The default box of the traditional SSD algorithm is calculated by the corresponding convolutional layer, which is the key to completing the image detection task. The calculation process of the default box is as follows. Firstly, the *min*_*size* and *max*_*size* of each layer’s FM are calculated. The *min*_*size* is shown in Eq (1).


$$min\_size=min\_dim*10/100+min\_size_{a}$$ (1)

In Eq (1), *min*_*dim* is the minimum input dimension. *min*_*size*_*a*_ is the minimum size of the upper output. The *max*_*size* is shown in Eq (2).


$$\max\_size=min\_dim*10/100+max\_size_{a}$$ (2)

In Eq (2), *max*_*size*_*a*_ 为D is the maximum size of the upper output. In Eqs (1) and (2), the value of *min*_*dim* is 300. The default initial values of *min*_*size*_*a*_ and *max*_*size* are 0. The values of *min*_*size*_*a*_ and *max*_*size* after the equal sign are previous calculation results. After all layers are calculated, the dimensions of the square and rectangular preset boxes for each layer can be further calculated. The side length of the smaller square box is the corresponding layer *min*_*size*_*a*_. The value *width*_*s*_ of the larger square box is calculated according to Eq (3).


$$width_{s}=\sqrt{min\_size\times max\_size}$$ (3)

For rectangular boxes, the length *width*_*r*_ is calculated according to Eq (4).


$$width_{r}=min\_size/{\left(aspect\_ratio\right)}^{1/2}$$ (4)

In Eq (4), *aspect*_*ratio* is the parameter that needs to be trained. The width *height*_*r*_ of the rectangular box is calculated, as shown Eq (5).


$$height_{r}=min\_size\times aspect\_ratio^{1/2}$$ (5)

By inverting the rectangular box, another rectangular box can be obtained. After calculation, the size of the preset boxes for each effective feature layer in the SSD structure can be obtained. Thus, the preset boxes for SSD target detection algorithms are filtered on each effective FM for subsequent operations.

When processing the redundant detection boxes generated by each FM, the SSD algorithm uses a non maximum suppression algorithm for filtering. The basic idea of the non maximum suppression algorithm is to select the detection box with the highest score in the local area. Then, the detection frame within the neighborhood that does not meet the set Intersection over Union (IoU) threshold is eliminated. The purpose is to solve an object being detected multiple times [26]. In the ideal scene of each real box, there is a preset box that completely overlaps with it [27]. If there is no such preset box, then a preset box with highest overlap is matched according to the IoU principle [28]. Assuming there are two predetermined rectangular boxes C1 and C2, the corresponding IoU calculation method is shown in Eq (6).


$$IoU=(area(C_{1}\cap C_{2}))/(area(C_{1}\cup C_{2}))$$ (6)

In Eq (6), *area*(*C*_1_∩*C*_2_) and *area*(*C*_1_∪*C*_2_) respectively represent the intersection area and union area of these two rectangular preset boxes.

From the above analysis of traditional SSD, in the feature extraction network part, SSD algorithm can predict vehicles with different sizes by using multi-scale FMs output from multiple convolution layers, improving the detection accuracy. However, in the actual traffic environment, there are interferences such as backgrounds. Due to the shallow convolutional structure limitation of SSD networks, the convolutional kernel size is relatively single. Therefore, multiple factors lead to insufficient extraction ability for target features. Therefore, in the generated FM, the semantic information of the target vehicle is insufficient, which makes it difficult to detect small target vehicles in practical applications. In response to this issue, the network structure of the SSD object detection algorithm is optimized.

**Fig 1:**
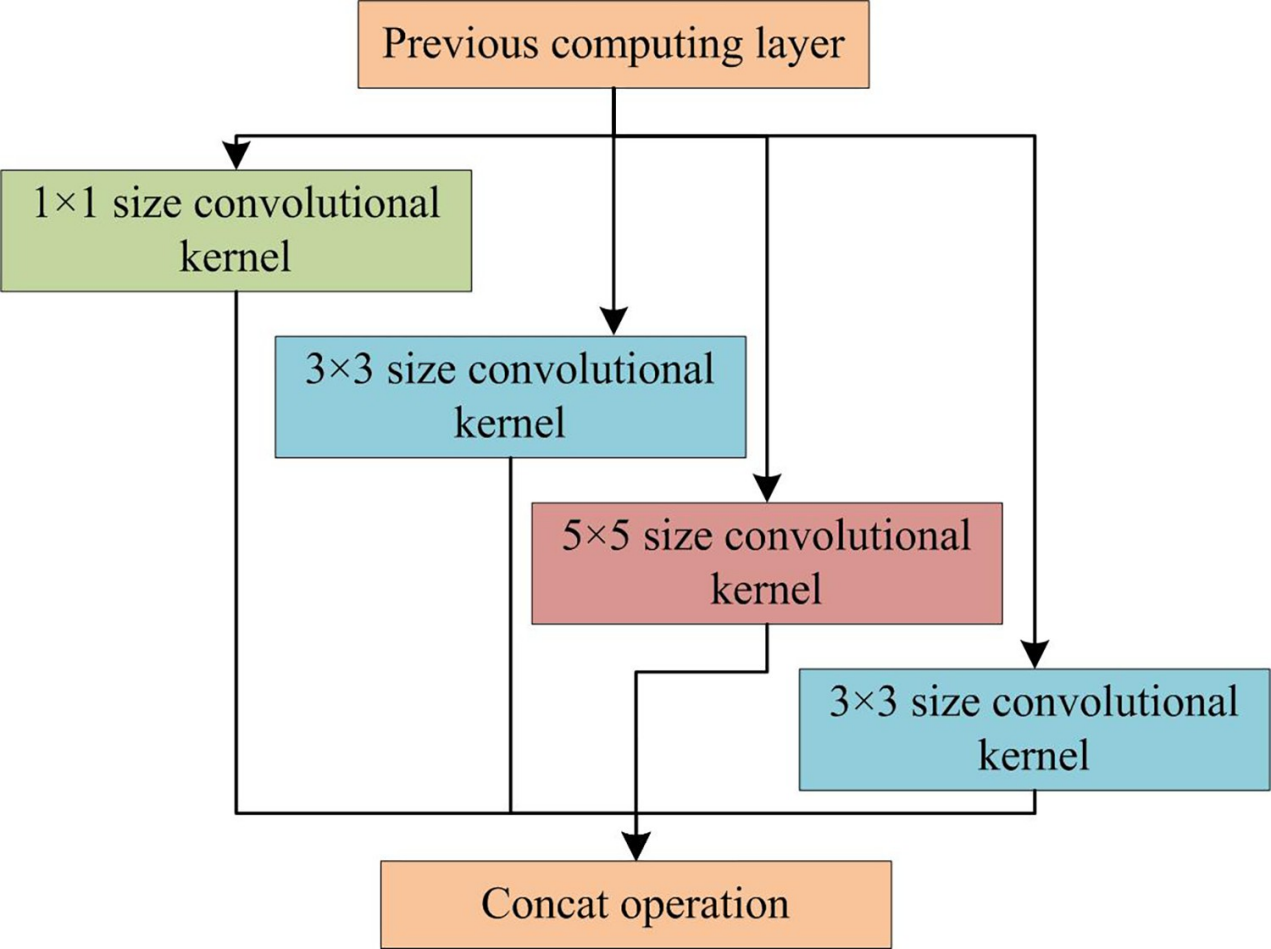
Inception structure of traditional SSD algorithm.

Based on past experience, increasing the depth and width of the network is the main method to enhance the feature extraction ability of the backbone network. However, continuously increasing the depth of convolutional networks can lead to a sharp increase in the network parameters, thereby reducing computational power. At the same time, it may lead to over fitting during the training process. The Inception structure is introduced to address these issues. The Inception introduces multiple convolutional kernels with different scales, namely 1×1, 3×3 and 5×5. These three different convolutional kernels optimize the convolutional structure of the network, greatly reducing the parameters in the original network and improving computational power. The structure is shown in Fig 1.

**Fig 2:**
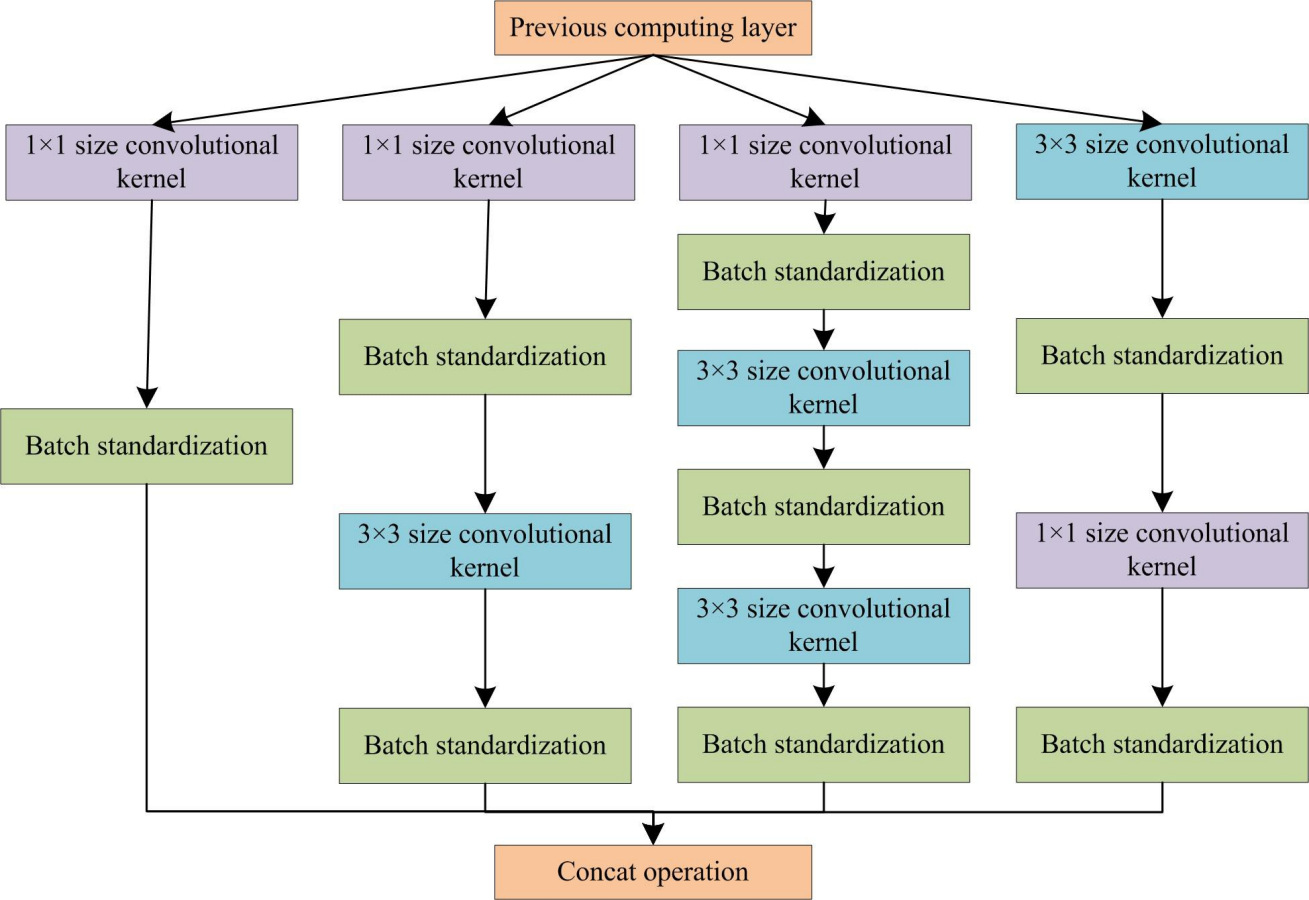
Schematic diagram of the improved inception structure.

The convolutional kernel of 5×5 requires a large amount of computation. Therefore, based on the original Inception structure, the idea of factorization is adopted. Two 3×3 convolutional kernels are used to replace 5×5 in the original structure. This further reduces the parameters involved in model operations and reduces the number of FMs generated at each layer, thereby accelerating computation speed. After the convolution operations of 1×1 and 3×3, batch standardization operations are added separately to ensure consistent data distribution through the convolution layer, making the model easier to converge and stabilize during training. The adjusted convolution structure is shown in Fig 2.

**Fig 3:**
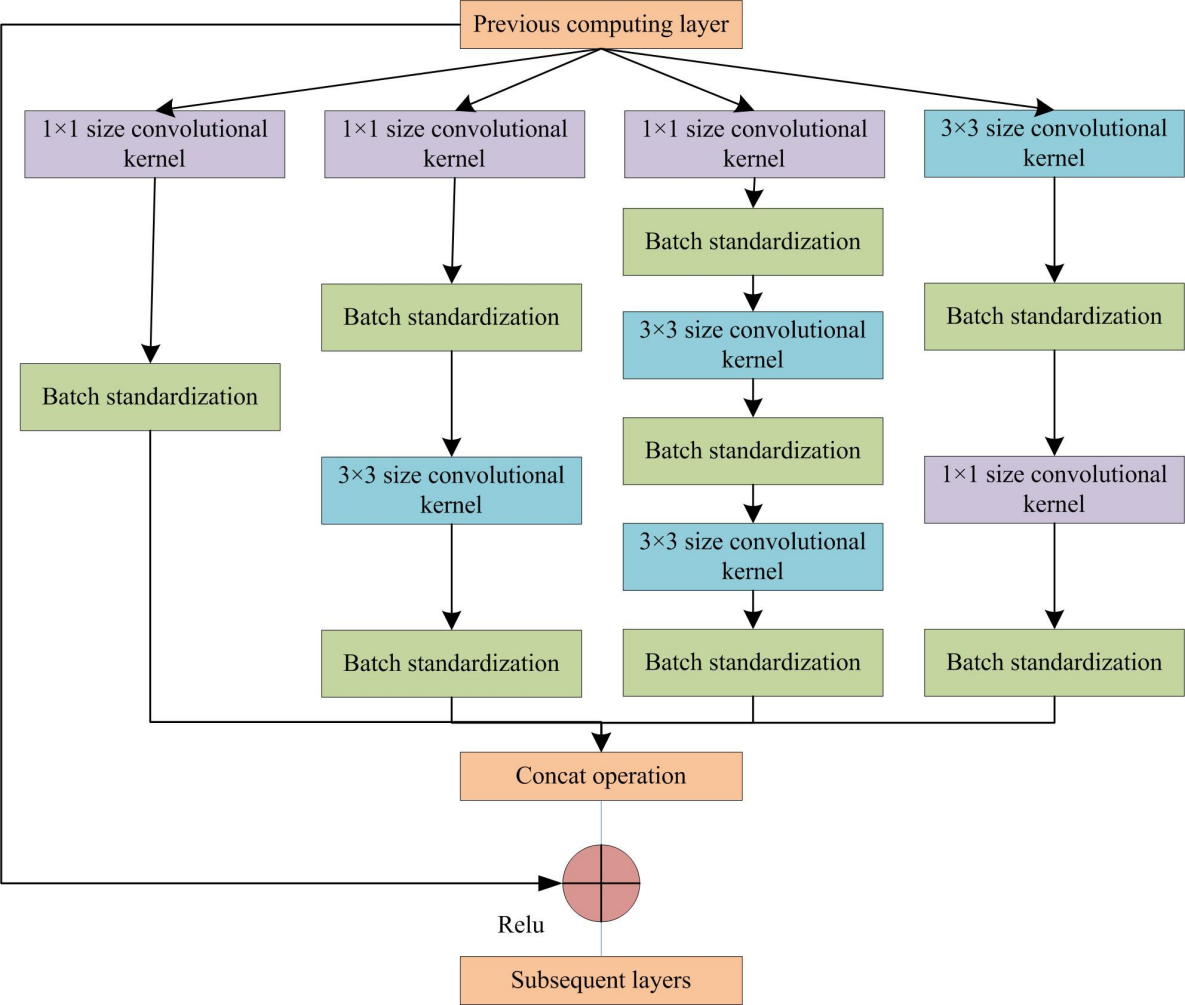
Improved inception network structure.

Assuming the size of the input FM is 38×38×512, for the Inception structure shown in Fig 2, it needs to pass through the four convolutional channels in the figure from left to right before outputting. The output result of the FM still meets the requirements of 512 layers. Therefore, the first channel data in this network needs to pass through a channel with the size of 128 1×1-convolutional kernels on the far left. The input size is 38×38×128. The second channel data needs to be dimensionally reduced to 38×38×128 through 128 1×1-convolution kernels. Then, through 256 3×3-convolution kernels, the output is 38×38×256. In the third channel, the data needs to go through 16 convolutions of 1×1, 32 convolutions of 3×3, and 64 convolutions of 3×3. The output is 38×38×64. After the fourth channel data is maximally pooled by 3×3 (pad = 1), 64 1×1-convolution kernels are performed. The output FM is 38×38×64. By adding the convolution results of the four parts, the channels in the FM remains unchanged at 512. After calculation, it can be concluded that the parameters of the overall convolution section are reduced from 38×38×512 = 184832 to 8856, which greatly reduce the number of parameters. After introducing this structure, the depth of the feature extraction network is increased. However, the increase in depth can also bring certain problems. To further optimize the network structure and alleviate the slow training speed caused by increasing network depth, the improved Inception network structure also introduces the idea of residual connections. By introducing a branch that directly connects the input and output, combined with other convolutional branches, the gradient dissipation and parameter non-update during the back propagation process of the deep network is avoided. At the same time, it can also accelerate the training speed. The improved Inception network structure incorporating residual connection ideas is shown in Fig 3.

When processing FMs, the convolutional FMs contain multi-channel information, but not all channel information is related to target detection. To enhance the effective channel information of FMs and improve the semantic expression ability, the channel AM-SENet module is introduced. The network structure of SENet is mainly consists of three stages, namely squeezing, excitation, and weighting. The first step in SENet calculation is to perform a squeezing operation on the FM. The FM is globally averaged and pooled. The FM with scale *w*×*h* and channel number *c* is compressed to the FM, with scale of 1×1 and the channel number of *c*. The calculation method is shown in Eq (7).


$$z_{c}=F_{sq}(u_{c})=1/(H\cdot W)*\sum_{i=1}^{H}\sum_{j=1}^{W}u_{c}(i,j)$$ (7)

In Eq (7), the output of the squeezing operation is obtained by global average pooling of the FM. *H* and *W* stand for the height and width of the FM, respectively. *u*_*c*_(*i*,*j*) stands for the pixels in row *i* and column *j* of the *c*-th channel in the FM. The output of the extrusion operation is set to *z*_*c*_. The incentive operation consists of two fully connected layers. The compressed features are treated as *c*-dimensional vectors. The vector is first dimensionally reduced to 1/*c* through a fully connected layer. Then, activated by the ReLU function, a new fully connected layer is connected to restore to the *c* dimension. Finally, through the sigmoid function, the weight values corresponding to all channels are obtained. The calculation process of the "incentive" operation is shown in Eq (8).


$$S_{c}=\sigma (g(w*z))=\sigma (w_{2}*\delta (w_{1}*z))$$ (8)

In Eq (8), σ stands for the Sigmaid function. δ stands for the ReLU function. *w*_1_ and *w*_2_ stand for the weights of the fully connected layer. *S*_*c*_ is the result obtained from the incentive operation. The final step is the weighting operation. The weight values obtained from the incentive operation are multiplied by the FM *u* to update the FM. The calculation process of the "weighted" operation is shown in Eq (9).


$${\tilde{x}}_{c}=u_{c}\cdot s_{c}x$$ (9)

In Eq (9), *u*_*c*_ is the FM. *s*_*c*_ is the weight value obtained from the incentive operation. ${\tilde{x}}_{c}$ is the result obtained from the weighted operation.

**Fig 4:**
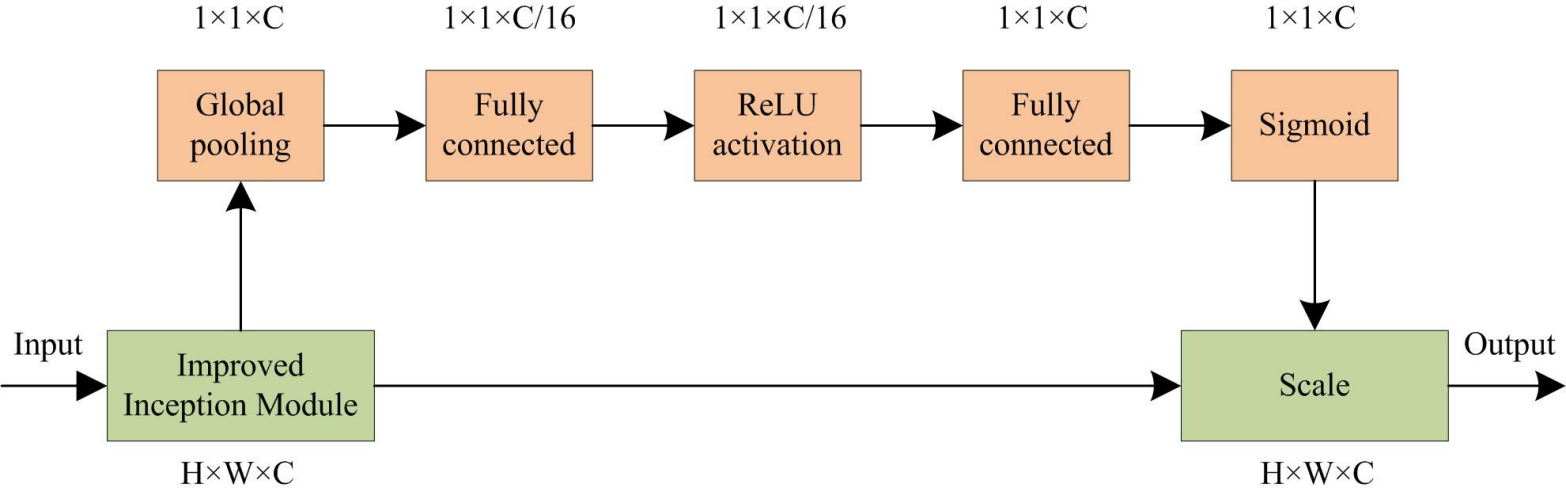
Insertion structure integrated into SENet.

After adding the SENet model to the Inception structure, the two FMs 38×38×512 and 19×19×1024 obtained from the convolution operation are connected to the SENet module. The Inception structure at this time is displayed in Fig 4. The content marked above the structural module in Fig 4 is the size data of the corresponding structure.

**Fig 5:**
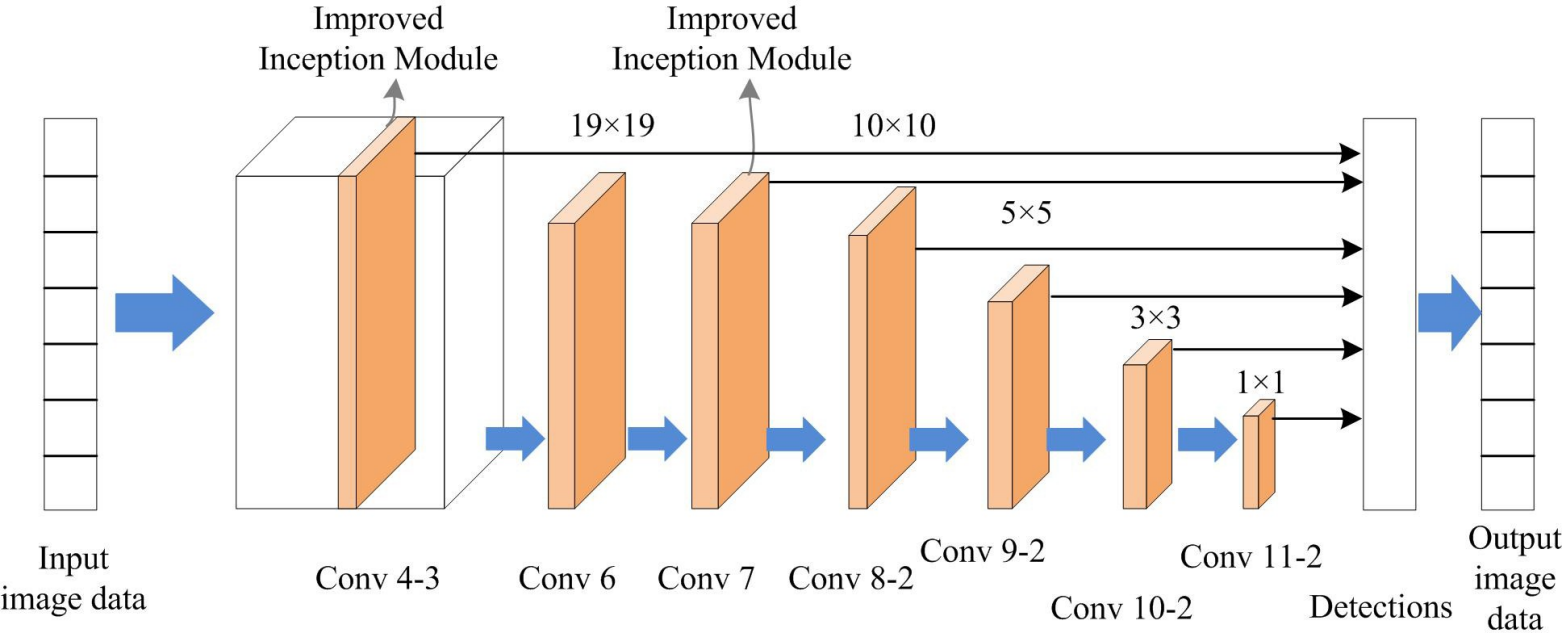
The network structure of the improved SSD algorithm.

The squeeze operation of SENet is performed using global pooling. Then, a bottleneck structure is constructed using two fully connected layers to obtain the weight information of each channel. This process involves two steps, dimensionality reduction and dimensionality increase. It has the following advantages. Firstly, the non-linear features of the network are added to better adapt to complex correlations between channels. Secondly, the reducing dimensionality first and then increasing dimensionality method significantly reduces the parameters and calculations involved in the calculation process. The overall network structure of the improved SSD is illustrated in Fig 5.

### 3.2 Vehicle flow detection model based on hybrid DeepSort and improved SSD

The purpose of improved SSD algorithm in image data processing is to improve the subsequent vehicle detection accuracy. The vehicle detection work is completed using the DeepSort algorithm. This algorithm is a commonly used method in the vehicle target detection industry and has stable performance, which is also the main reason for choosing this algorithm to build a traffic flow detection model. DeepSort applies the deep learning concept, mainly using CNN to obtain target characteristics. In the target tracking stage, the innovative measurement method of cosine distance is combined with Markov distance to more effectively connect the predicted position with the detected position. The cascading matching method is used to improve the global allocation problem while dealing with the frequent switching of target unique numbers. The DeepSort algorithm is mainly composed of three calculation modules: feature extraction based on CNN, feature matching, and target position prediction. The focus of the DeepSort algorithm is on designing the CNN structure, which has a significant impact on the overall image recognition accuracy of the algorithm.

The application scenario of traffic flow detection requires rapid display for real-time measurement results. Therefore, the overall complexity of the model should not be too high. To reduce the computational complexity of the CNN module in the DeepSort algorithm, FMs are compressed. The down sampling of CNN pooling layer is calculated based on Eq (10).


$$X_{j}^{l}=f(\beta_{j}^{l}down(X_{i}^{l-1})+b_{j}^{l})$$ (10)

In Eq (10), $X_{j}^{l-1}$ stands for the output of the *j*-th neuron in the *l*−1-th layer. $b_{j}^{l}$ is the bias coefficient of the corresponding neuron. *f*_*n*_(⋅) is the mapping function. *down*(⋅) refers to the maximum and average of the current layer’s feature values. To obtain better training stability, the two norm Loss function *loss* is selected to construct CNN. The calculation method is shown in Eq (11).


$$loss=\sum_{n=1}^{N}{\left\|Y_{n}-\tilde{Y}(X_{n})\right\|}^{2}$$ (11)

In Eq (11), $\tilde{Y}(X_{n})$ stands for the prediction result. *X*_*n*_ is the input data. To prevent over fitting in the neural network, regularization terms need to be added. The commonly used regularization methods include one norm regularization *L*_*re*1_ and two norm regularization. *L*_*re*1_ is displayed in Eq (12).


$$loss_{re1}=loss+\lambda \|\theta \|$$ (12)

In Eq (12), *θ* is the parameter to be optimized. *λ* is the weight attenuation coefficient. If the parameter is not 0, the regularization term in the second norm can suppress the parameter size. It can also avoid the model being too sparse. Therefore, the binomial regularization term is adopted to design the neural network. The calculation method is shown in Eq (13).


$$loss_{re2}=loss+\lambda {\left\|\theta \right\|}^{2}$$ (13)

Finally, the training mode of CNN is designed. The objective function is *L*. The optimal network parameter is θ*, as shown in Eq (14).


$$\theta^{*}=\arg\;\underset{\theta }{\min}L$$ (14)

The gradient descent is applied to optimize the network. In the calculation, the derivative of *L* is first taken to obtain the gradient. Then, the gradient update model is used for updating until it completes convergence, as illustrated in Eq (15).


$$\theta_{j+1}=\theta_{j}+\frac{lr\cdot \partial L}{\partial \theta_{j}}$$ (15)

**Fig 6:**
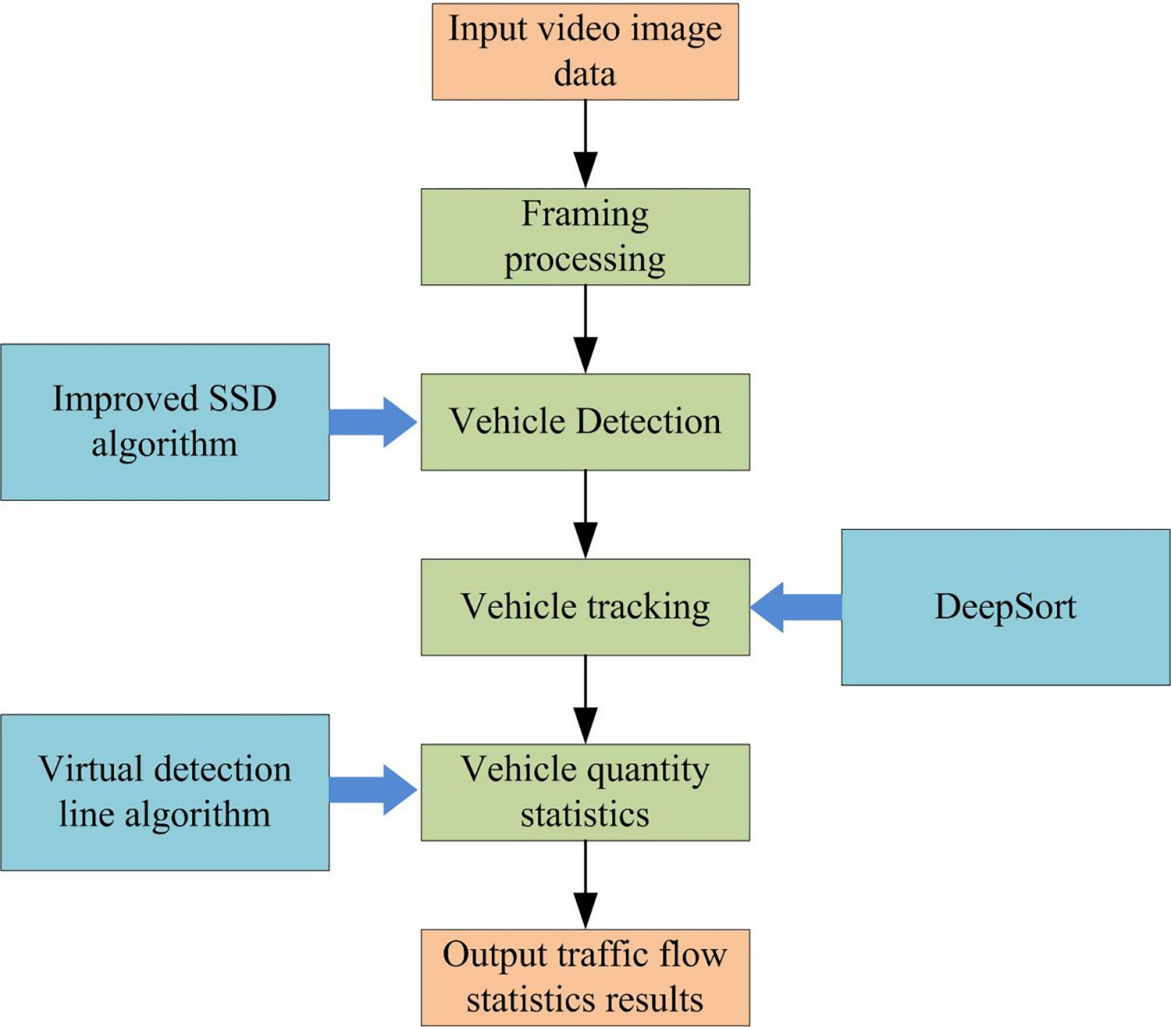
Vehicle flow detection model based on improved SSD and DeepSort algorithm.

From this, a vehicle flow detection model based on improved SSD and DeepSort can be obtained. Fig 6 illustrates the computational procedure.

When using improved SSD and DeepSort algorithm for traffic flow statistics, some common counting problems still need to be solved. Specifically, when counting passing vehicles on a single virtual detection line (VDL), factors such as vehicle missed detections and frame dropouts can lead to missing vehicle statistics. Therefore, the concept of dual virtual detection bands is proposed to collect traffic flow information, which can accurately determine the vehicle traveling direction in different lanes simultaneously.

**Fig 7:**
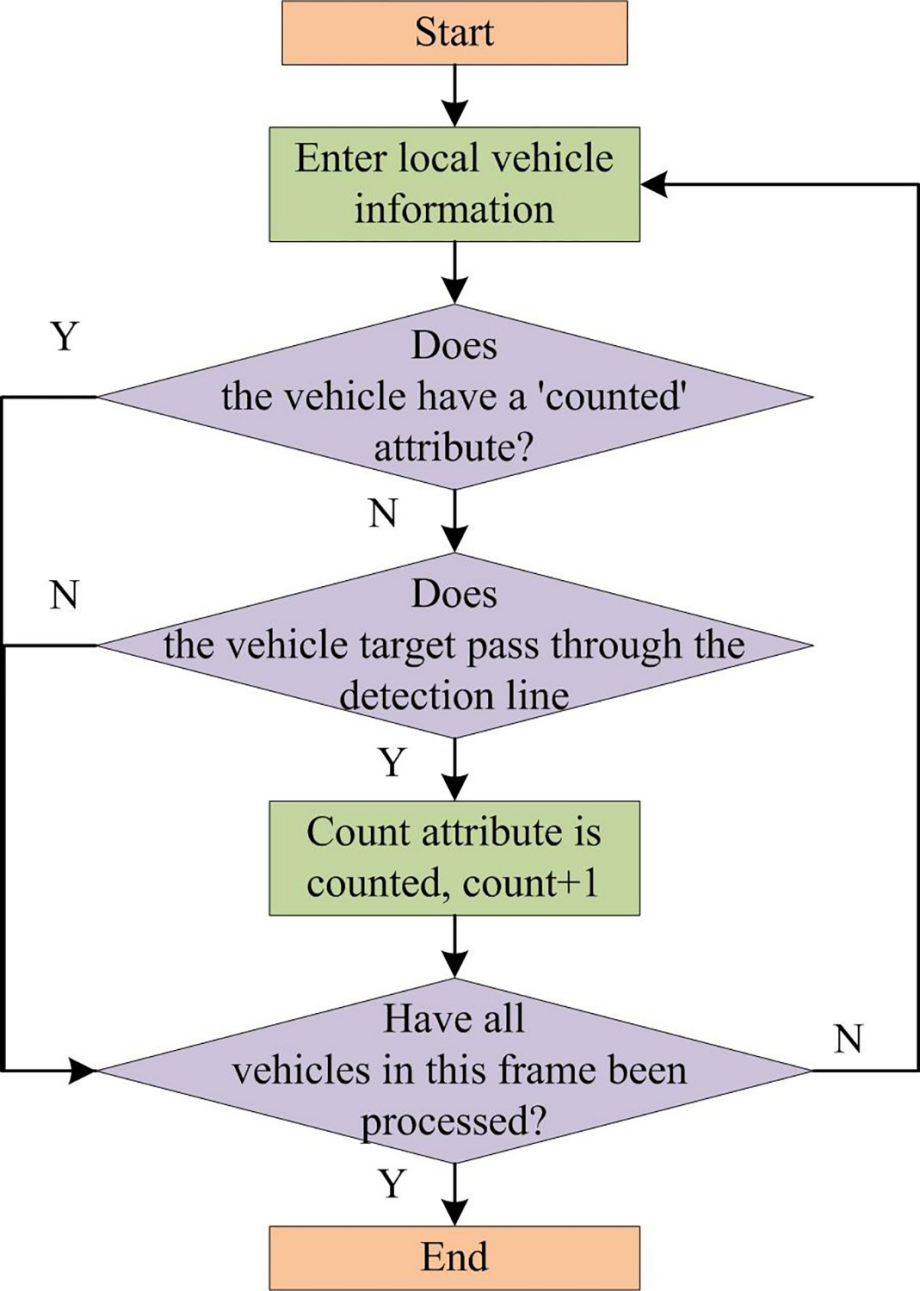
Judgment process of vehicle flow statistics method based on virtual detection line.

The implementation principle of the vehicle flow statistics method based on virtual detection VDL is as follows. At the center of the video image, which is perpendicular to the lane line, a VDL is set. The detected and tracked vehicles cross this detection line. When the vehicle tracking box intersects with the detection line, it is considered that there are vehicles passing by, and the number of vehicles increases by 1. When all vehicles pass, the traffic flow statistics task is finally completed. The judgment process of the vehicle flow statistics method based on VDL is shown in Fig 7. In Fig 7, to identify different vehicles, all vehicles in a frame of image are tracked by the DeepSort algorithm. Each detected and successfully tracked vehicle is assigned a unique identity code. To facilitate whether all vehicles should be counted, a counting attribute is set for each vehicle. In the counting rule, 1 represents that it is counted, and 0 represents that it is not counted. After the vehicle passes the detection line, the counting attribute is set to 1, and there will be no repeated counting thereafter. If the attribute value of the vehicle count in the previous frame is 0, then it will be judged again in the current frame whether the vehicle tracking box intersects with the detection line. If it is successfully detected, the count attribute value of the vehicle is 1. This process will continue until all vehicles in the current frame are processed.

Relying solely on one virtual detection line for counting may result in vehicle statistics being missed due to vehicle missed detections, frame drops, and other reasons. It is impossible to avoid some problems, such as incomplete detection, frame dropout, or duplicate tracking. Therefore, the virtual detection line is improved. Unlike the virtual detection line algorithm, the improved method sets a green and red virtual detection strip with a certain width above and below the center of the video image. If the vehicle collision point appears within the detection zone area, it will be counted once. This is to prevent the situation where vehicles near the virtual detection line are not detected. In addition, the improved method can accurately perceive the vehicle direction based on the order in which the vehicle’s collision points pass through the red and green detection strips. It can be well applied to more operating conditions on two-way roads.

## 4. Performance verification test of vehicle flow detection model

Two experiments are designed to verify the availability of the improved SSD and traffic flow detection model designed in this research. In the two experiments, accuracy (ACC), precision (PRE), recall (REC), detection speed, and Loss function value are used as test indicators. In the experiment, Python programming language is used to build various algorithms. They are run in the deep learning framework Tensor-flow.

### 4.1 Experimental plan design

The hardware environment for the experiment is as follows. The computing computer is HP Shadow Genie 4. The running memory is Samsung brand. The size is 16GB, the storage hard disk is 1024GB, and the central computing unit is Intel 5–8300. In the performance testing experiment of the improved SSD algorithm, the algorithms involved in the calculation include RestNet50, Faster RCNN, SSD300, and Improved SSD300 (ISSD300) constructed based on SSD300. The parameters of all models are determined by multiple adjustments within the established range of the target detection industry. The parameter scheme of ISSD300 designed in this research is illustrated in Table 1.

**Table 1:** ISSD300 parameter setting scheme.

Number	Name	Value Or Mode	Number	Name	Value Or Mode
#01	Batch size	64	#06	Does the hidden layer have an offset term	Y
#02	Iterations	700	#07	Weight attenuation	0.0002
#03	Momentum coefficient	0.90	#08	Number of RNN units in BLSTM single chain	4
#04	Neuron loss rate	0.40	#09	Parameter initialization method	Random initialization
#05	Learning rate	Training 1–100 times: 0.1; 101~300 training sessions: 0.01; 301–700 training sessions: 0.001	#10	Training methods	Graphics Processing Unit

In the experiment of vehicle flow detection model, manual statistics, traditional SSD algorithm combined with DeepSort algorithm (SSD+DeepSort), improved SSD combined with DeepSort algorithm (ISSD+DeepSort), and You Only Look Once version 3 (YOLOv3) algorithm are selected to construct the detection model. The reason for choosing YOLOv3 instead of YOLOv8 in this study is as follows. The latter adds more model clusters to the computational structure. Therefore, compared with YOLOv3, the model complexity and computational speed are significantly reduced. This is not conducive to the application of the algorithm in traffic vehicle detection. The statistical personnel in the manual statistical plan are 10 auxiliary police officers with rich vehicle identification experience from across the country. The datasets used in the two experiments are recorded by surveillance cameras on roads in a certain city in China. The VeRidataset is obtained by frame processing. 12734 images containing different types and numbers of vehicles were randomly selected as the dataset. Among them, 30% of the dataset, which is 3820 images, is used for the testing, while the rest is the training set. The dataset contains vehicle objects such as cars, trucks, buses, and fire trucks. The video generation time includes morning, noon, afternoon, evening, and midnight. The shape of vehicle images includes side, front, and back. In addition, before training each model, some preprocessing is required on the dataset, mainly to delete videos with missing pixels, unify video pixel sizes of different sizes, and enhance data with insufficient clarity. Firstly, images with poor quality in the original dataset are removed, such as blurry, overexposed, or completely devoid of vehicles. The second step is diversity enhancement, ensuring that the dataset covers different weather conditions (sunny, rainy, foggy), different times (day, night), and different traffic densities. If there is a lack of corresponding types of images, they need to be filtered and supplemented in the complete dataset. The third step is image enhancement, applying image processing techniques to adjust image brightness and contrast, and using high/low pass filters to improve image quality. This helps the model to better identify vehicles in practical environments. The final step is to check the consistency of annotations. If the dataset has been annotated (such as the location and type of vehicles), the consistency and accuracy of all annotations need to be ensured.

### 4.2 Performance analysis of improved SSD algorithm

**Fig 8:**
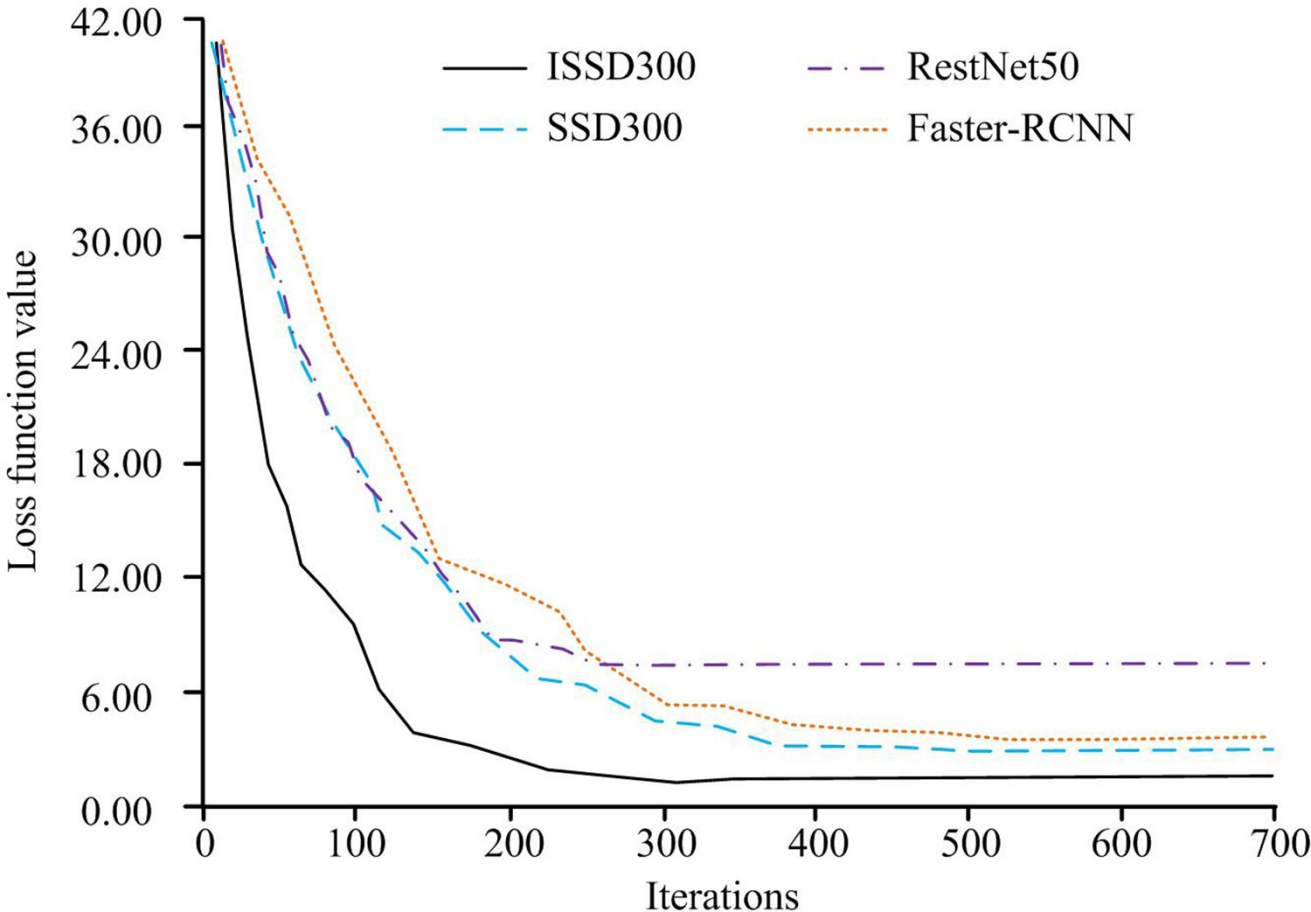
Loss function values of target detection algorithms during training.

The test results are analyzed in a modular manner. Firstly, the target detection algorithms are compared and analyzed, and then the overall detection methods are analyzed. Moreover, ablation experiments are added to the comparison of each module. The unimproved original algorithm comparison model is added to comprehensively and scientifically compare the performance of design methods. The Loss function value change of each target detection algorithm in the training is shown in Fig 8. The horizontal axis (HA) and vertical axis (VA) in Fig 8 respectively represent the iterations in the training and the Loss function value of each algorithm. The line style is used to distinguish between different algorithms. In Fig 8, with the increase of the iterations, the Loss function of each algorithm has a downward trend and gradually converges. From the perspective of convergence speed, the ISSD300 algorithm, which is designed and mixed with the improved Inception module, is significantly faster than that of SSD300. Moreover, the convergence speed is the fastest among all the comparison algorithms. Among the other three algorithms, SSD300 is similar to Faster RCNN in convergence speed, and RestNet50 is relatively fast in convergence. The Loss function values after convergence of ISSD300, SSD300, Faster RCNN and RestNet50 algorithms are 1.25, 2.61, 2.68 and 8.14 respectively.

**Fig 9:**
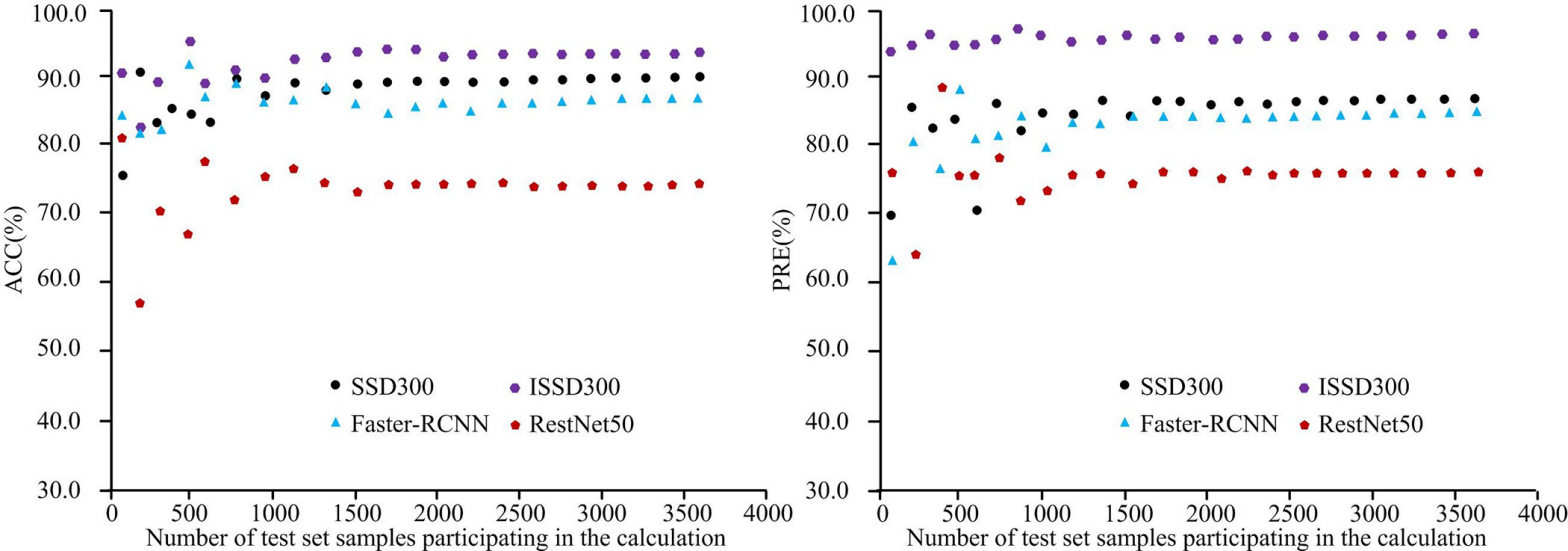
Comparison of ACC and PRE of various algorithms on the test set.

After the training is completed, each target recognition algorithm is input into different numbers of test set images. The ACC and PRE values of each algorithm are compared, as displayed in Fig 9. The HA in Fig 9 displays the test set samples participating in the calculation. The VA of sub-graph and sub-graph respectively display the ACC and PRE. In Fig 9, the two indicator values of each algorithm show a decreasing fluctuation pattern as the test set images participating in the calculation increases. This is mainly because the calculation sample is small. Some difficult to recognize images have a significant impact on the overall detection accuracy. When the test sample is a complete set of 3820 road vehicle monitoring images, the ACC values of ISSD300, SSD300, Faster RCNN, and RestNet50 algorithms are 93.6%, 89.2%, 87.3%, and 74.0%, respectively. The PRE values of ISSD300, SSD300, Faster RCNN, and RestNet50 algorithms are 96.0%, 85.4%, 83.2%, and 74.5%, respectively. The improved SSD algorithm has better car image detection capability.

In the complete test set, the target detection speed of different vehicle models in various algorithms is compared. The models involved in the comparison include sedans, trucks, motorcycles, off-road vehicles, tricycles, construction vehicles, and fire trucks. Table 2 illustrates the statistical results. In Table 2, from the perspective of vehicle type, the detection speed of each algorithm for motorcycle images is significantly slower than other algorithms. The motorcycle appearance differs greatly from other vehicles. The differences in appearance between different types of motorcycles are also significant, resulting in poor learning performance and slower recognition speed for such images. From the algorithmic perspective, the RestNet50 algorithm has the fastest overall speed in different vehicle image recognition, followed by ISSD300. The average detection speeds of ISSD300, SSD300, Faster RCNN, and RestNet50 for various types of vehicles are 26.3ms/piece, 21.0ms/piece, 22.9ms/piece, and 27.7ms/piece, respectively.

**Table 2:** Comparison of detection speeds of various algorithms on various vehicles (ms/pie).

Number	Vehicle type	ISSD300	SSD300	Faster-RCNN	RestNet50
#1	Sedan	28	22	24	30
#2	Truck	28	23	22	32
#3	Motorcycle	20	15	17	25
#4	Off-road vehicle	27	24	23	24
#5	Tricycle	26	20	25	28
#6	Engineering construction vehicle	28	21	24	29
#7	Fire truck	27	22	25	26

### 4.3 Performance test results analysis of vehicle flow detection model

**Fig 10:**
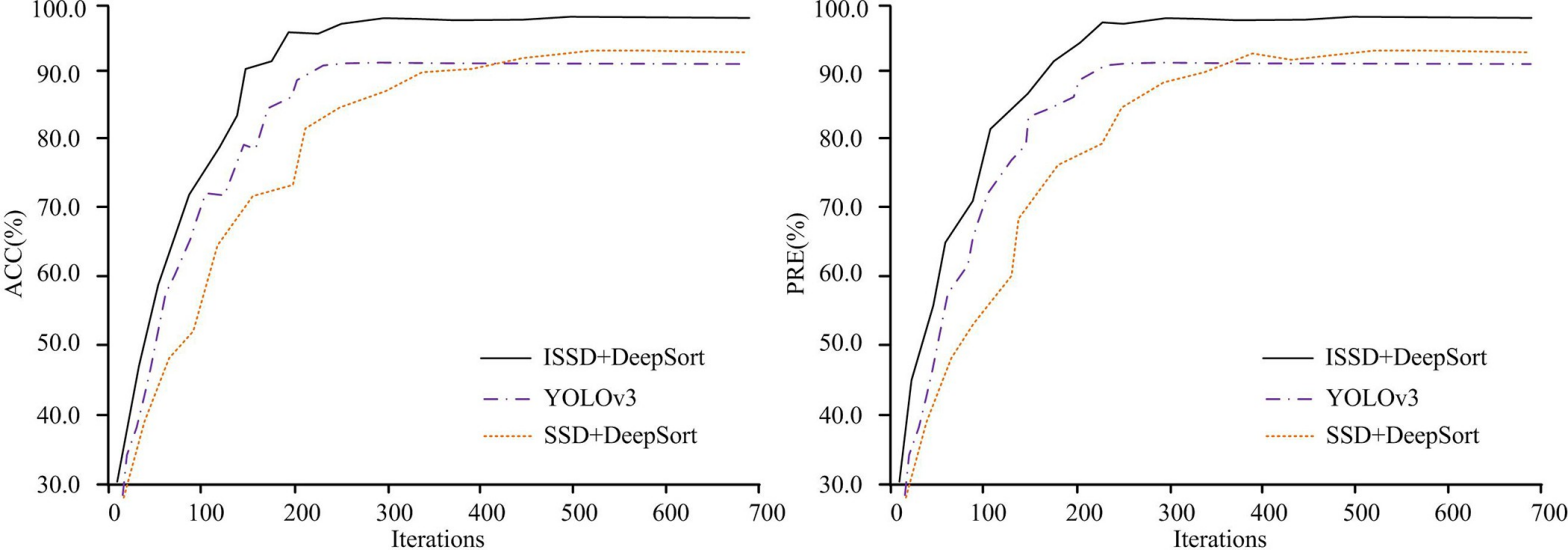
Comparison of ACC and PRE among statistical models on the training set.

In the performance testing experiment of the vehicle flow detection model, the statistical result changes in ACC and PRE values are shown in Fig 10. The HA in Fig 10 stands for the iterations, while the VA in sub-graph and sub-graph represent the indicators ACC and PRE, respectively. The line style is used to distinguish different statistical models. The manual statistical method does not require training, so it is not included in Fig 10. In Fig 10, as the training progresses, the statistical accuracy of each model gradually improves and converges to a certain value. This indicates that the parameter settings of each model are relatively reasonable. When the number of iterations reaches 700, all statistical models are completed training. The ACC and PRE of ISSD+DeepSort, SSD+DeepSort, and YOLOv3 models are 98.4%, 91.5%, 89.7%, and 98.2%, 91.3%, and 89.5%, respectively. Moreover, the test results show that occluded vehicles can also be accurately identified, which is due to the stronger feature extraction ability of the algorithm designed this time. The image of the occluded vehicle is not subjected to additional processing in the experiment. This issue is not separately considered in the study. By comparing the recognition results of various methods, the designed method still performs better than the comparison model on vehicles with occlusion.

**Fig 11:**
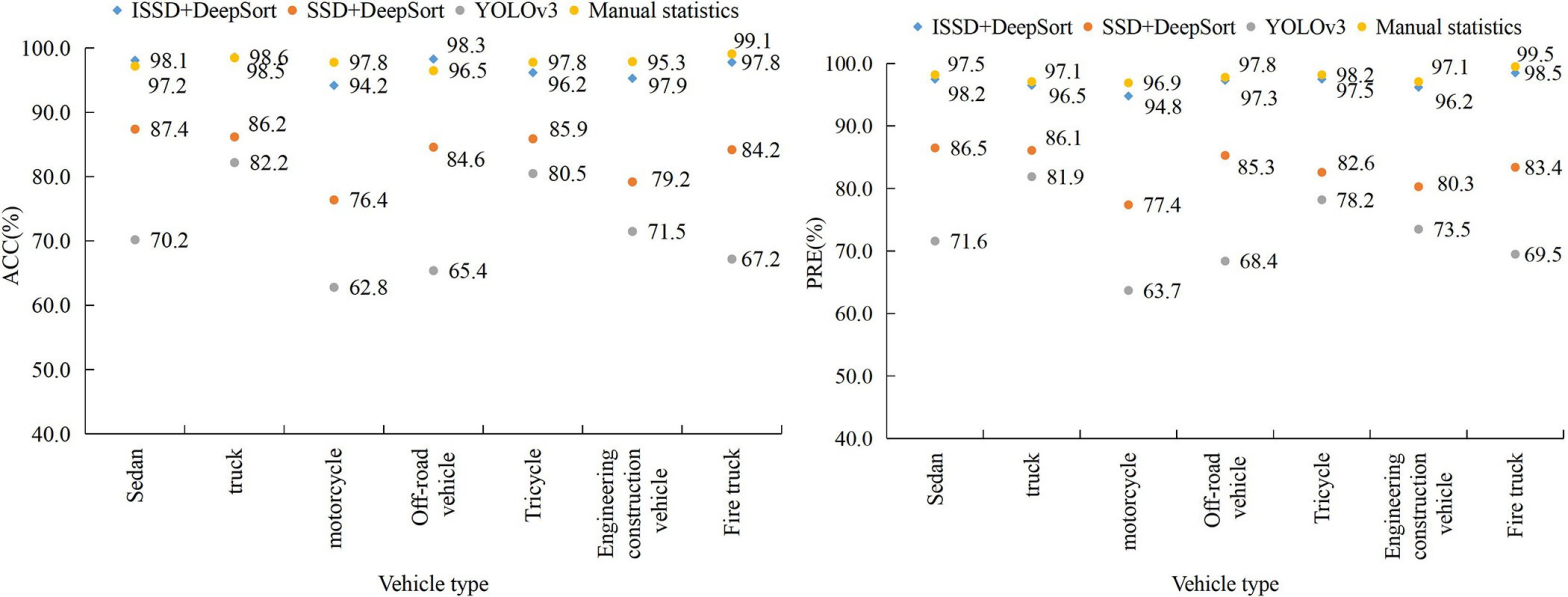
Comparison of ACC and PRE among statistical models on the test set.

After the training is completed, the ACC and PRE statistical results of each model on the test set are illustrated in Fig 11. The HA in Fig 11 displays the types of cars in various test sets. The meaning of the VA is consistent with Fig 10. Different icons represent different traffic flow statistical models. In Fig 11, the designed ISSD+DeepSort statistical model has the smallest difference in accuracy compared to manual statistics. Except for manual statistics, all methods have the lowest statistical accuracy on motorcycles. The average ACC of various images for ISSD+DeepSort, SSD+DeepSort, and YOLOv3 models are 96.9%, 83.4%, and 71.4%, respectively. The average PRE of various images for ISSD+DeepSort, SSD+DeepSort, and YOLOv3 models are 96.8%, 83.1%, and 72.4%, respectively. To ensure the scientificity and reliability of the experimental results, all experiments in Fig 11 were repeated for reliability analysis. The reliability coefficients of ISSD+DeepSort, SSD+DeepSort, and YOLOv3, as well as manual statistical methods, were calculated to be 0.864, 0.859, 0.913, and 0.882, all higher than 0.800, indicating good consistency in this experiment.

**Fig 12:**
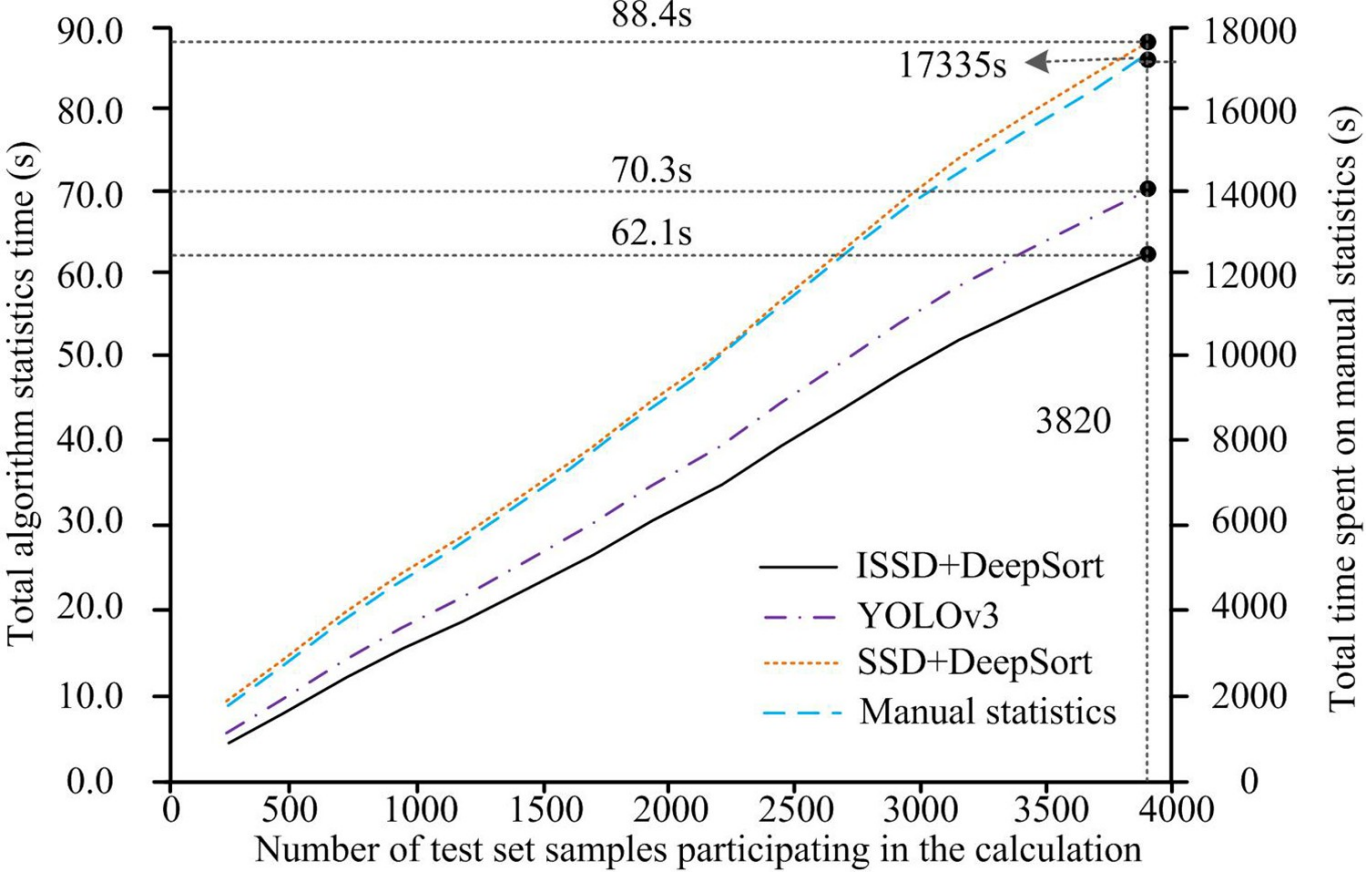
Total computational time of each statistical model under different test image numbers.

The total calculation time of each statistical model under different test image numbers is depicted in Fig 12. The HA in Fig 12 represents the test sample size for different schemes. The left VA stands for the total computational time. The right VA stands for the total statistical time of manual statistical methods. In Fig 11, as the test images increases, the statistical time of each model and method shows an almost linear growth pattern. It indicates that the calculation speed of each model varies slightly, and the model has good operational stability. From the perspective of time-consuming data, the statistical time of manual statistical methods is much higher than that of statistical models constructed by other intelligent algorithms. The difference reaches three orders of magnitude. In the intelligent algorithm statistical model, the ISSD+DeepSort model designed for research has the least computational time and the highest computational efficiency. This is because the improved Inception module that builds this model greatly reduces the computational complexity. The total statistical time for ISSD+DeepSort, SSD+DeepSort, YOLOv3, and artificial models on the overall test set is 62.1s, 88.4s, 70.3s, and 17335s, respectively.

**Fig 13:**
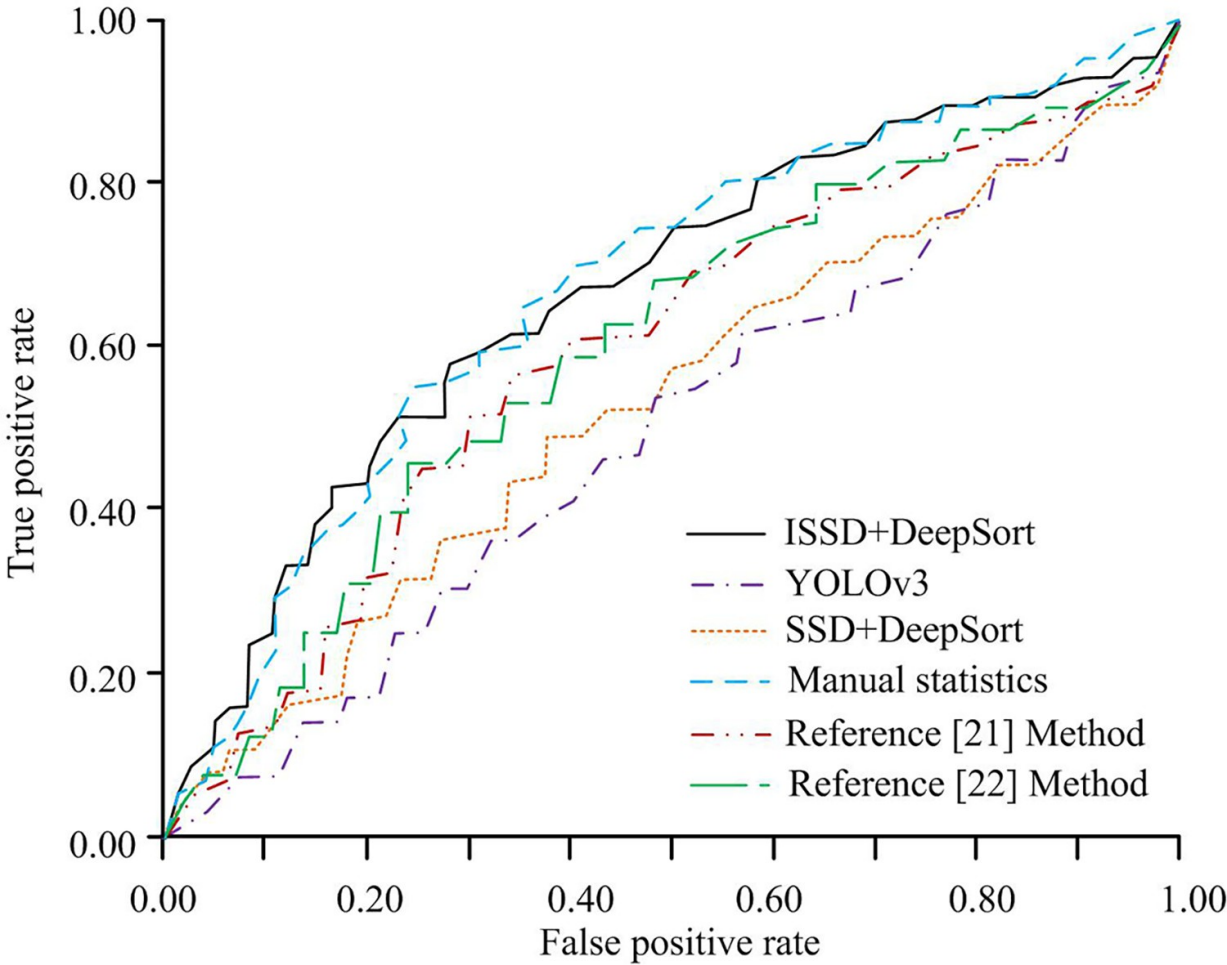
AUC values of ROC for each traffic flow statistical model on the test set.

Finally, the area under curve (AUC) values of the receiver operating characteristic curve (ROC) are compared. The statistical results are shown in Fig 13. The HA in Fig 13 stands for the false positive rate. The VA stands for the true positive rate. To improve the reliability of the research results, references [21, 22], which have also designed vehicle recognition models, are also included for comparison. In Fig 13, the corresponding AUC values for ISSD+DeepSort, SSD+DeepSort, YOLOv3, artificial models, reference [21] Method and reference [22] Method are 0.71, 0.56, 0.54, 0.72, 0.63 and 0.62, respectively. The AUC value of the model designed in this study is higher than that of other comparative models. The vehicle flow detection and classification ability are the best.

## 5. Conclusion

Aiming at the poor performance of the vehicle flow detection model in the intelligent transportation system, an intelligent urban road traffic flow detection model based on the enhanced SSD is designed. Two experiments are designed to verify the performance of the improved SSD target detection algorithm and the intelligent model. According to the findings, the ISSD300 algorithm designed with the improved Inception module has the fastest convergence speed. The Loss function values of ISSD300, SSD300, Faster RCNN and RestNet50 algorithms after convergence are 1.25, 2.61, 2.68 and 8.14 respectively. When the test sample is a complete set of 3820 road vehicle monitoring images, the ACC values of ISSD300, SSD300, Faster RCNN, and RestNet50 algorithms are 93.6%, 89.2%, 87.3%, and 74.0%, respectively. The PRE values of ISSD300, SSD300, Faster RCNN, and RestNet50 algorithms are 96.0%, 85.4%, 83.2%, and 74.5%, respectively. The improved SSD algorithm has better car image detection capability. The traffic flow statistics show that when the number of iterations reaches 700, all statistical models have completed training. The ACC of ISSD+DeepSort, SSD+DeepSort, and YOLOv3 models were 98.4%, 91.5%, and 89.7%, respectively. The ACC of ISSD+DeepSort, SSD+DeepSort, and YOLOv3 models were 98.2%, 91.3%, and 89.5%, respectively. After the training is completed, except for manual statistics, all methods have the lowest statistical accuracy on motorcycles. The average ACC of various images for ISSD+DeepSort, SSD+DeepSort, and YOLOv3 models were 96.9%, 83.4%, and 71.4%, respectively. The average PRE of various images for ISSD+DeepSort, SSD+DeepSort, and YOLOv3 models are 96.8%, 83.1%, and 72.4%, respectively. The ISSD+DeepSort model has the least computational time and the highest computational efficiency. This is because the improved Inception module that builds the model significantly reduces the computational complexity of the overall model. The total statistical time for ISSD+DeepSort, SSD+DeepSort, YOLOv3, and artificial models on the overall test set is 62.1s, 88.4s, 70.3s, and 17335s, respectively. The corresponding AUC values for ISSD+DeepSort, SSD+DeepSort, YOLOv3, and artificial models are 0.71, 0.56, 0.54, and 0.72, respectively. The experimental data proves that the urban road traffic flow detection model based on the improved SSD algorithm has excellent detection ability. However, the drawback is that no larger scale data is selected for testing. This is also an area for improvement in subsequent research.

## Supporting information

S1 File (ZIP)

## References

[1] Chen H., Gao J., Jiang X., Gao Z., and Zhang W., "Optimization-Inspired Deep Learning High-Resolution Inversion for Seismic Data," GEOPHYSICS, vol. 86, no. 3, pp. R265–R276, Jan. 2021. doi: 10.1190/geo2020-0034.1

[2] Zhao Q., Zhang B., Feng W., Du Z., Zhang H., and Sun D., "Long-term real time object tracking based on multi-scale local correlation filtering and global re-detection," COMPUTING, vol. 102, no. 6, pp. 1487–1510, Mar. 2020. doi: 10.1007/s00607-020-00807-8

[3] Gao Z., Liu Y., Xu G., and Wen X., “Pairwise Attention Network for Cross-Domain Image Recognition,” NEUROCOMPUTING, vol. 453, no. 1, pp. 393–402, Jan. 2021. doi: 10.1016/j.neucom.2020.06.147

[4] Yang Y. and Song X.,“Research on face intelligent perception technology integrating deep learning under different illumination intensities,” JCCE, vol. 1, no. 1, pp. 32–36, Jan. 2022.

[5] Deng L., Zhang J., Xu G., and Zhu H., "Infrared small target detection via adaptive M-estimator ring top-hat transformation," PR, vol. 112, no. 1, pp. 107729.1–107729.9, Nov. 2020. doi: 10.1016/j.patcog.2020.107729

[6] Gao C., Yan J., Peng X., and Liu H., "Signal structure information-based target detection with a fully convolutional network," INFORM SCIENCES, vol. 576, pp. 345–354, Oct. 2021. doi: 10.1016/j.ins.2021.06.066

[7] Rad M. K., and Andargoli S. M. H., "Power control and beamforming in passive phased array radars for low probability of interception," DIGIT SIGNAL PROCESS, vol. 117, no. 3, pp. 103165.1–103165.11, Oct. 2021. doi: 10.1016/j.dsp.2021.103165

[8] Wang K., and Liu M., "A feature-optimized Faster regional convolutional neural network for complex background objects detection," IET IMAGE PROCESS, vol. 15, no. 2, pp. 378–392, Feb. 2020. doi: 10.1049/ipr2.12028

[9] Zhang H., Peng G., Wu Z., Gong J., Xu D., and Shi H. Z.,“MAM: A multipath attention mechanism for image recognition,” IET IMAGE PROCESS, vol. 16, no. 1, pp. 1–10, Dec. 2022. doi: 10.1049/ipr2.12370

[10] Zhao H., Wu B., Guo Y., Chen G., and Ye D., "SSWS: An edge detection algorithm with strong semantics and high detectability for spacecraft," OPTIK, vol. 247, pp. 168037.1–168037.13, Dec. 2021. doi: 10.1016/j.ijleo.2021.168037

[11] Tao C., He H., Xu F., and Cao J., "Stereo priori RCNN based car detection on point level for autonomous driving," KNOWL-BASED SYST, vol. 229, Oct. 2021. doi: 10.1016/j.knosys.2021.107346

[12] Hu W. J., Xie T. Y., Li B. S., Du Y. X., and Xiong N. N.,“An Edge Intelligence-based Generative Data Augmentation System for IoT Image Recognition Tasks,” J INTERNET TECHNOL, vol. 22, no. 4, pp. 765–778, Jul. 2021. doi: 10.53106/160792642021072204005

[13] Dong Z., Ji X., Zhou G., Gao M. Y., and Qi D. L., "Multimodal neuromorphic sensory-processing system with memristor circuits for smart home applications," IEEE Trans. Ind. Appl., vol. 59, no. 1, pp. 47–58, July 2022. doi: 10.1109/TIA.2022.3188749

[14] Dong Z., Lai C. S., Zhang Z., Qi D. L., Gao M. Y., and Duan S. K., "Neuromorphic extreme learning machines with bimodal memristive synapses," Neurocomputing, vol. 453, pp. 38–49, May 2021. doi: 10.1016/j.neucom.2021.04.049

[15] Dong Z., Ji X., Lai C. S., Qi D. L., Zhou G. D., and Lai L. L., "Memristor-based hierarchical attention network for multimodal affective computing in mental health monitoring," IEEE Cons. Electron. Magazine, vol. 12, no. 4, pp. 94–106, July 2022. doi: 10.1109/MCE.2022.3159350

[16] Ji X., Lai C. S., Zhou G., Dong Z. K., Qi D. L., and Lai L. L., "A flexible memristor model with electronic resistive switching memory behavior and its application in spiking neural network," IEEE Trans. NanoBiosci., vol. 22, no. 1, pp. 52–62, February 2022. doi: 10.1109/TNB.2022.3152228 35171775

[17] Ji X., Qi D., Dong Z., Lai C. S., Zhou G. D., and Hu X. F., "TSSM: Three-State Switchable Memristor Model Based on Ag/TiO x Nanobelt/Ti Configuration," Int. J. Bifurcation Chaos, vol. 31, no. 7, p. 2130020, November 2021. doi: 10.1142/S0218127421300202

[18] Dong Z., Ji X., Lai C. S., Qi D. L., "Design and implementation of a flexible neuromorphic computing system for affective communication via memristive circuits," IEEE Commun. Magazine, vol. 61, no. 1, pp. 74–80, October 2022. doi: 10.1109/MCOM.001.2200272

[19] Ji X., Dong Z., Han Y., Lai C. S., Qi D. L., "A Brain-inspired Hierarchical Interactive In-memory Computing System and its Application in Video Sentiment Analysis," IEEE Trans. Circuits Syst. Video Technol., vol., doi: 10.1109/TCSVT.2023.3275708, May 2023.

[20] Ji X., Dong Z., Han Y., Lai C. S., Zhou G. D., Qi D. L., "EMSN: An Energy-Efficient Memristive Sequencer Network for Human Emotion Classification in Mental Health Monitoring," IEEE Trans. Consum. Electron., vol., doi: 10.1109/TCE.2023.3263672, March 2023.

[21] Rajput S. K., Patni J. C., Alshamrani S. S., Chaudhari V., Dumka A., Singh R., et al., "Automatic Vehicle Identification and Classification Model Using the YOLOv3 Algorithm for a Toll Management System," Sustainability, vol. 14, no. 15, pp. 9163, May 2022. doi: 10.3390/su14159163

[22] González-Cepeda J., Ramajo Á., Armingol J. M., "Intelligent video surveillance systems for vehicle identification based on multinet architecture," Information, vol. 13, no. 7, pp. 325, November 2022. doi: 10.3390/foods12224156

[23] Wang Y., Li S., Wu D., and Yan H., "Noninvasive prenatal testing of hereditary colorectal cancer syndromes using cell‐free DNA in maternal plasma," PRENATAL DIAGNOSIS, vol. 42, no. 5, pp. 557–566, May 2022. doi: 10.1002/pd.6137 35343616

[24] Gou X., Liu Q., Rong H., Hu M., Paul P., Deng F., et al, “A Novel Spiking Neural P System for Image Recognition,” INT J UNCONV COMPUT, vol. 16, no. 2/3, pp. 121–139, Jun. 2021.

[25] Hao M., Liu G., and Xie D., "Hyperspectral face recognition with a spatial information fusion for local dynamic texture patterns and collaborative representation classifier," IET IMAGE PROCESS, vol. 15, no. 8, pp. 1617–1628, Jun. 2021. doi: 10.1049/ipr2.12131

[26] Liu Y., Wang J., and Bai W., “Commodity Price Recognition and Simulation of Image Recognition Technology Based on the Nonlinear Dimensionality Reduction Method,” ADV MATH PHYS, vol. 2021, no. 4, pp. 1045342.1–1045342.9, Oct. 2021. doi: 10.1155/2021/1045342

[27] Gu B., and Ling C. X., "Generalized Error Path Algorithm," INT J PATTERN RECOGN, vol. 120, no. 1, pp. 108112.1–108112.12, Jun. 2021. doi: 10.1016/j.patcog.2021.108112

[28] Rosa G., Guillaud F., Priol P., and Renet J., "Parameter affecting the I3S algorithm reliability: how does correcting for body curvature affect individual recognition?," WILDLIFE RES, vol. 48, no. 1, pp. 38–43, Sep. 2020. doi: 10.1071/WR19238

