# Supplementary figures and images for: Traffic flow detection method based on improved SSD algorithm for intelligent transportation system
Source: PLoS One. 2024 Mar 14;19(3):e0300214. doi: 10.1371/journal.pone.0300214 (PMC10939265; doi:10.1371/journal.pone.0300214)

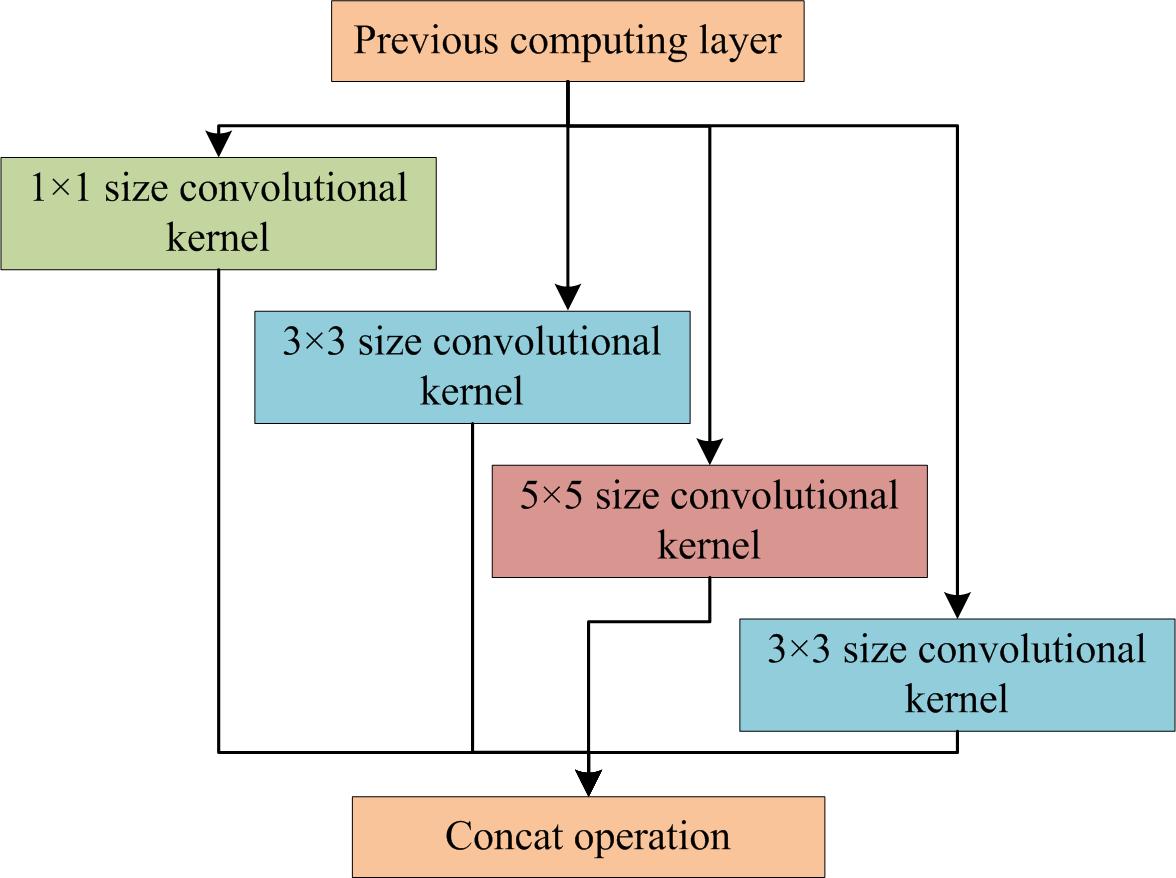

Supplement: S1 File — (ZIP) [file pone.0300214.s001.zip › Supporting information/Figure 1.jpg]

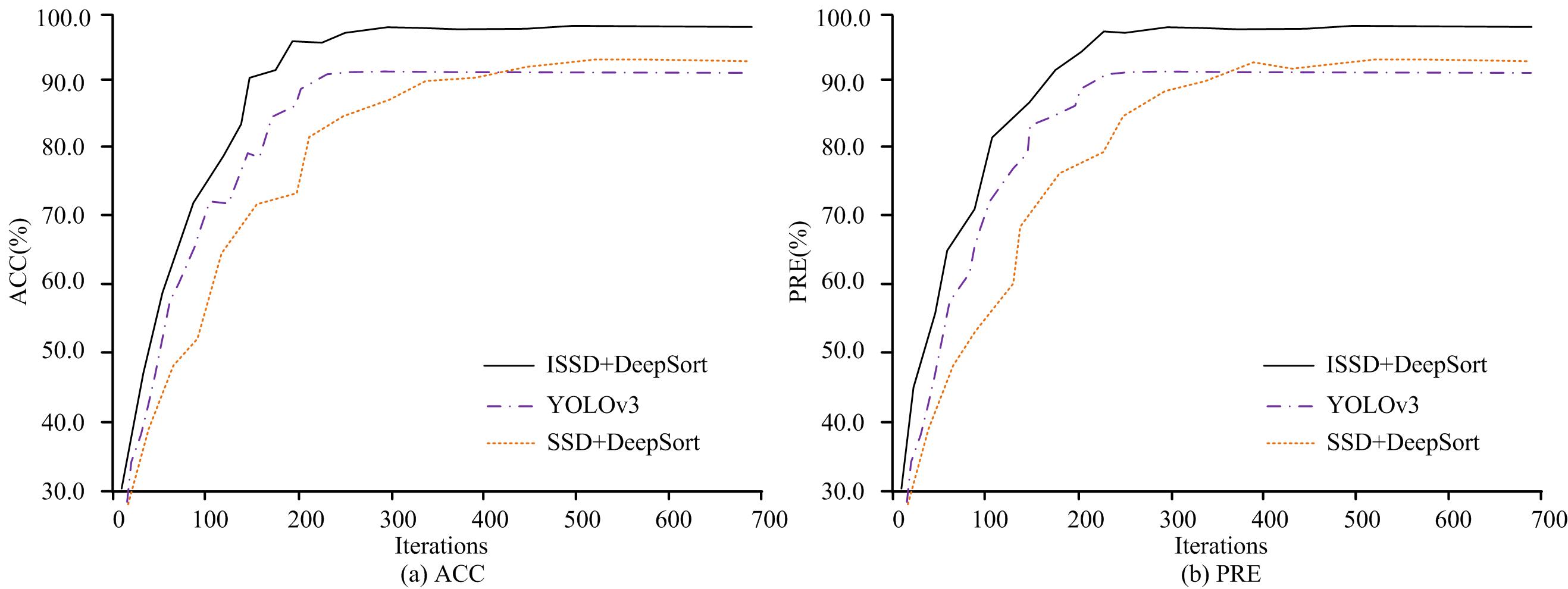

Supplement: S1 File — (ZIP) [file pone.0300214.s001.zip › Supporting information/Figure 10.jpg]

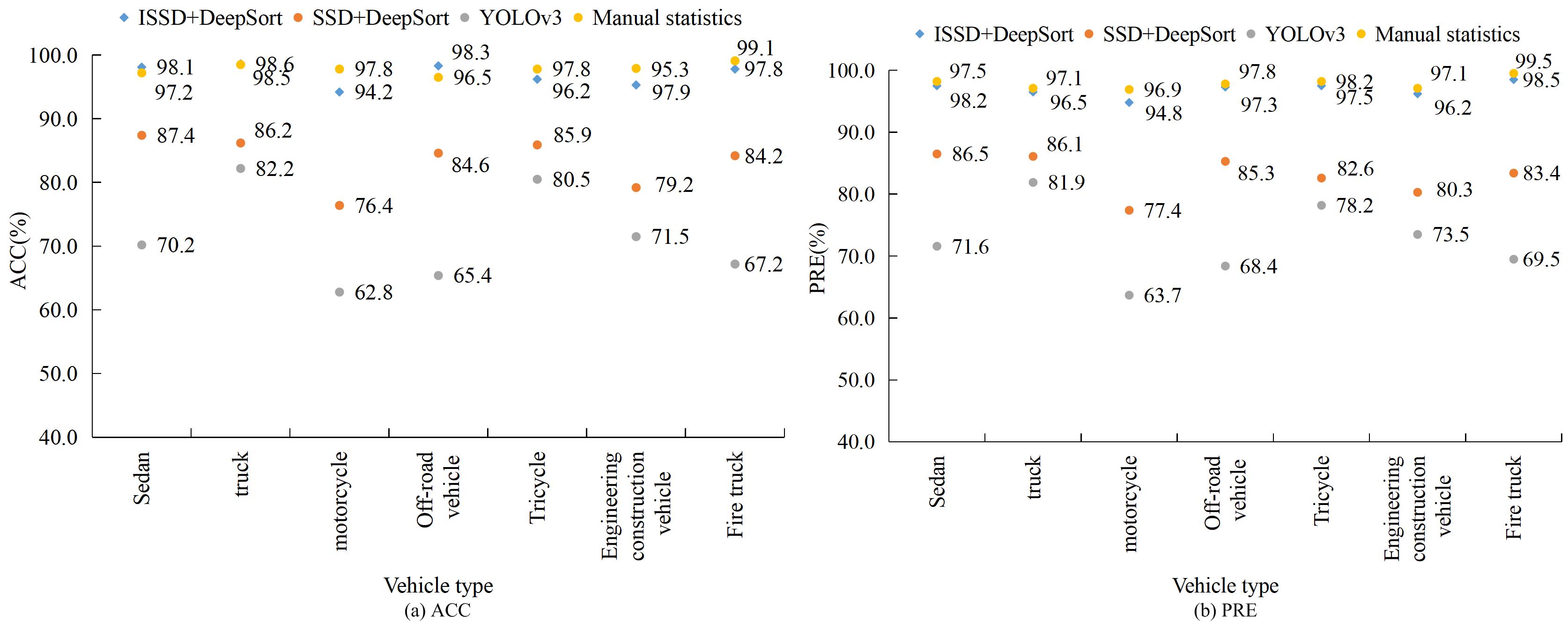

Supplement: S1 File — (ZIP) [file pone.0300214.s001.zip › Supporting information/Figure 11.jpg]

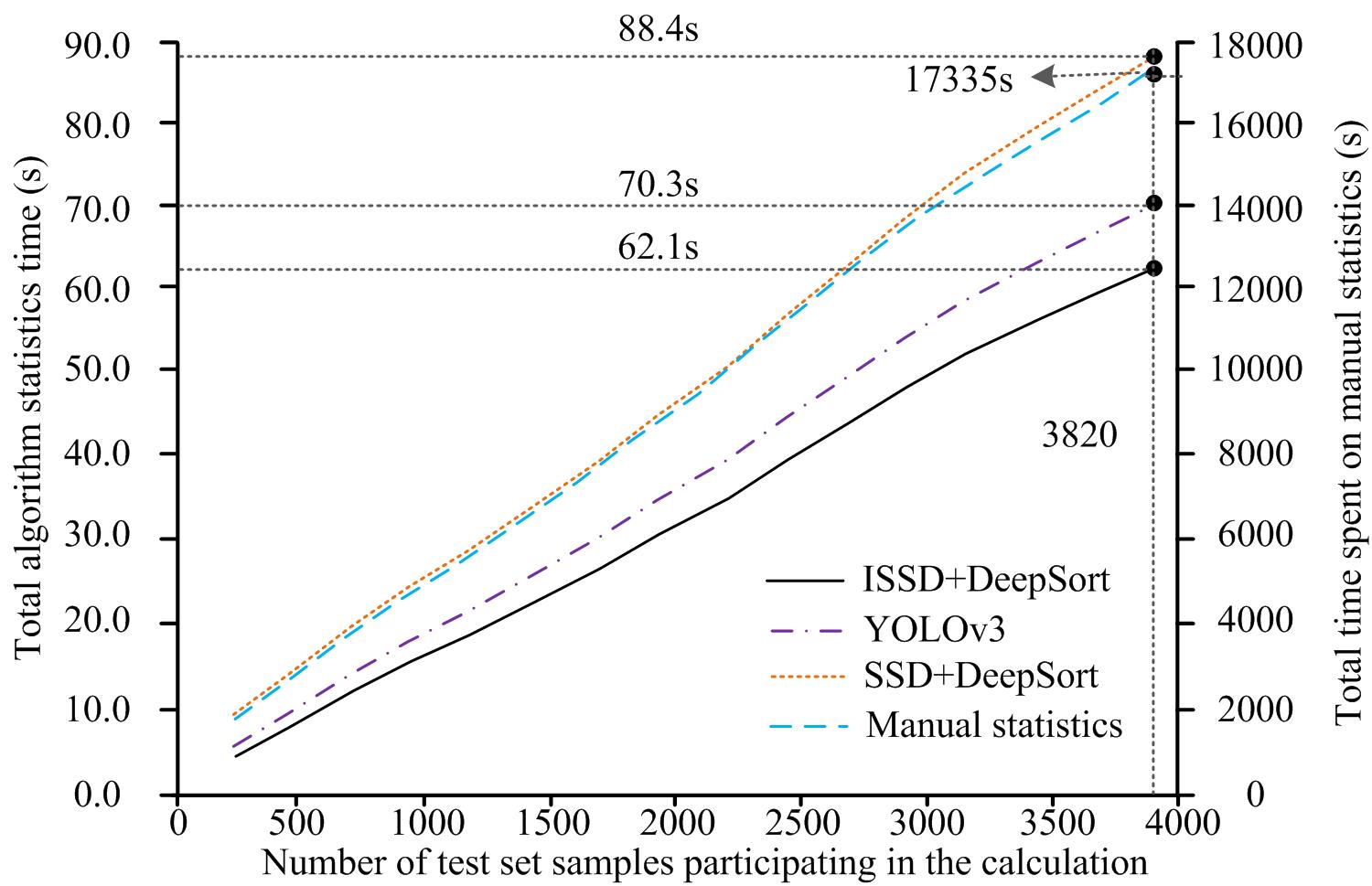

Supplement: S1 File — (ZIP) [file pone.0300214.s001.zip › Supporting information/Figure 12.jpg]

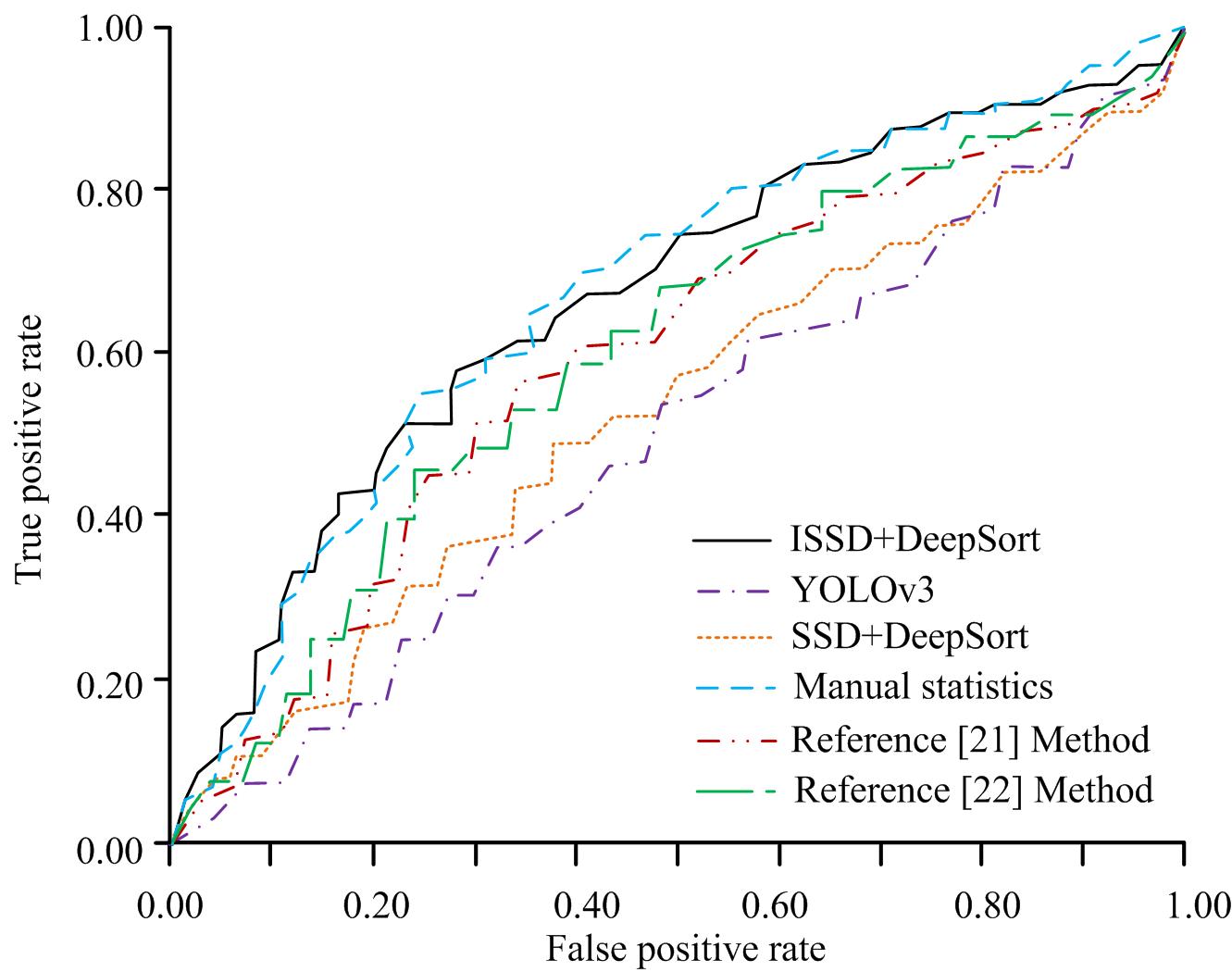

Supplement: S1 File — (ZIP) [file pone.0300214.s001.zip › Supporting information/Figure 13.jpg]

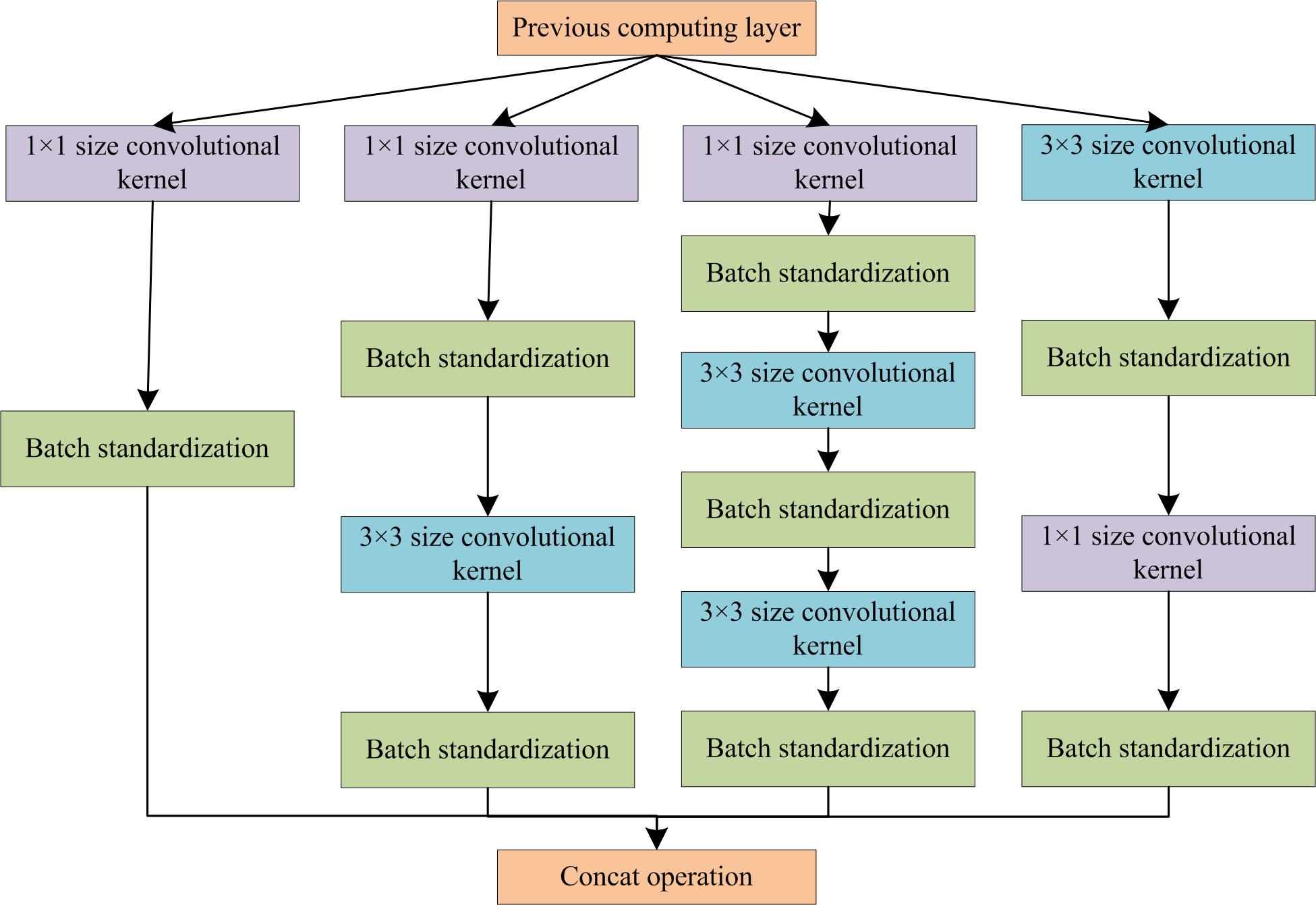

Supplement: S1 File — (ZIP) [file pone.0300214.s001.zip › Supporting information/Figure 2.jpg]

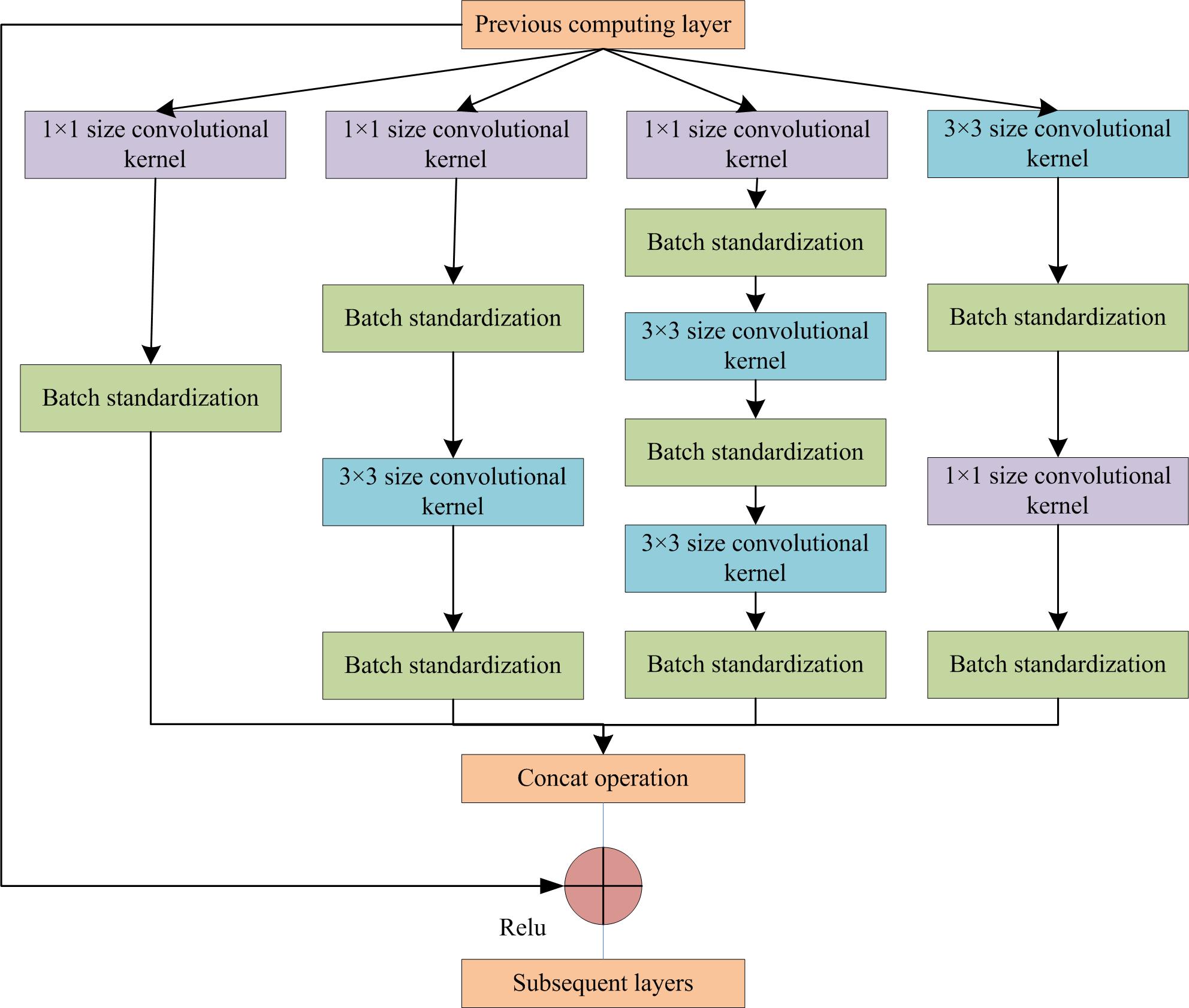

Supplement: S1 File — (ZIP) [file pone.0300214.s001.zip › Supporting information/Figure 3.jpg]

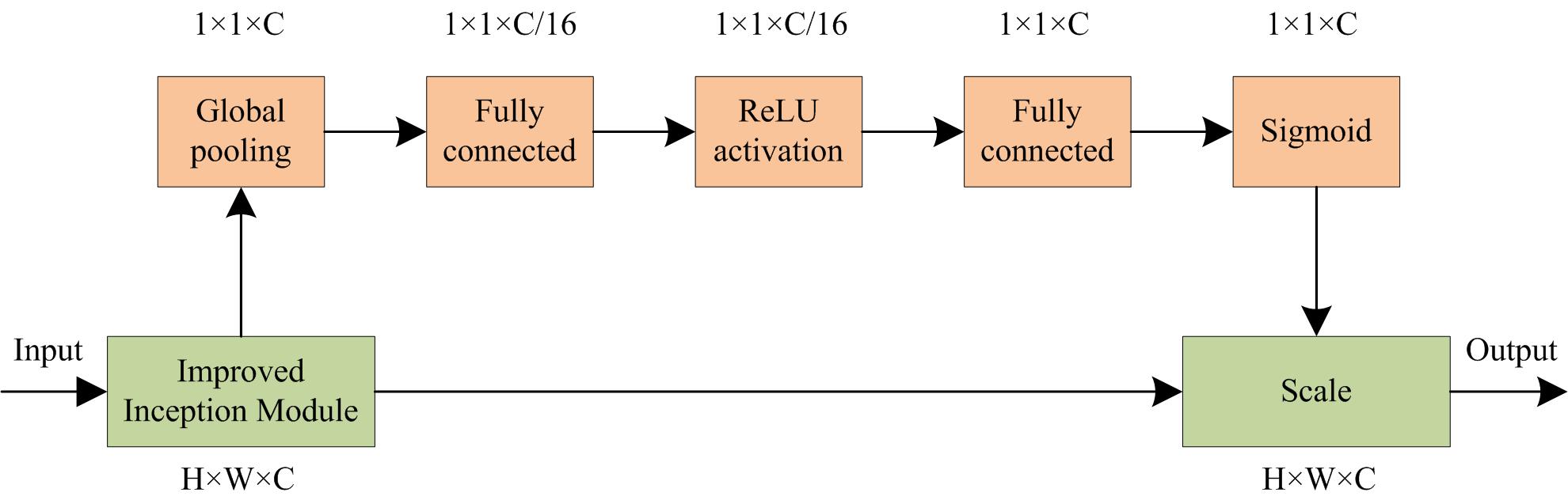

Supplement: S1 File — (ZIP) [file pone.0300214.s001.zip › Supporting information/Figure 4.jpg]

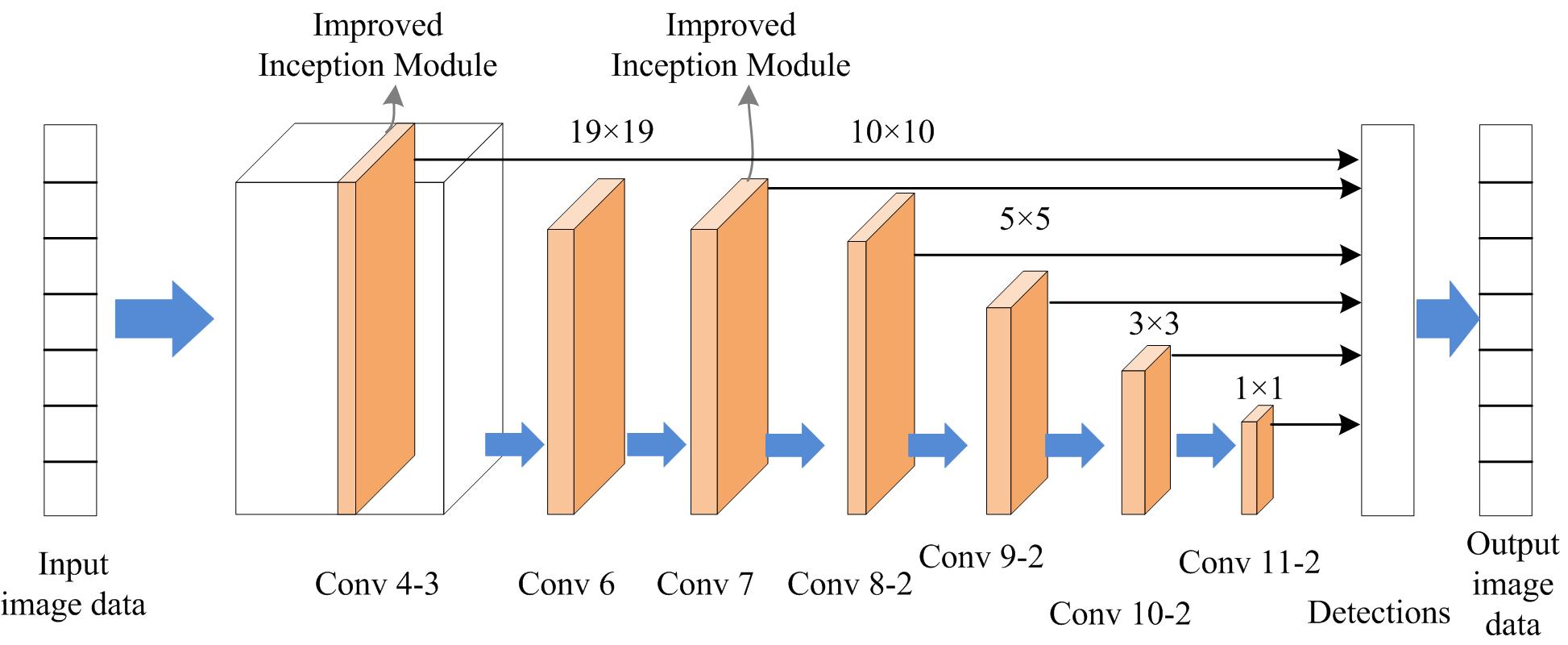

Supplement: S1 File — (ZIP) [file pone.0300214.s001.zip › Supporting information/Figure 5.jpg]

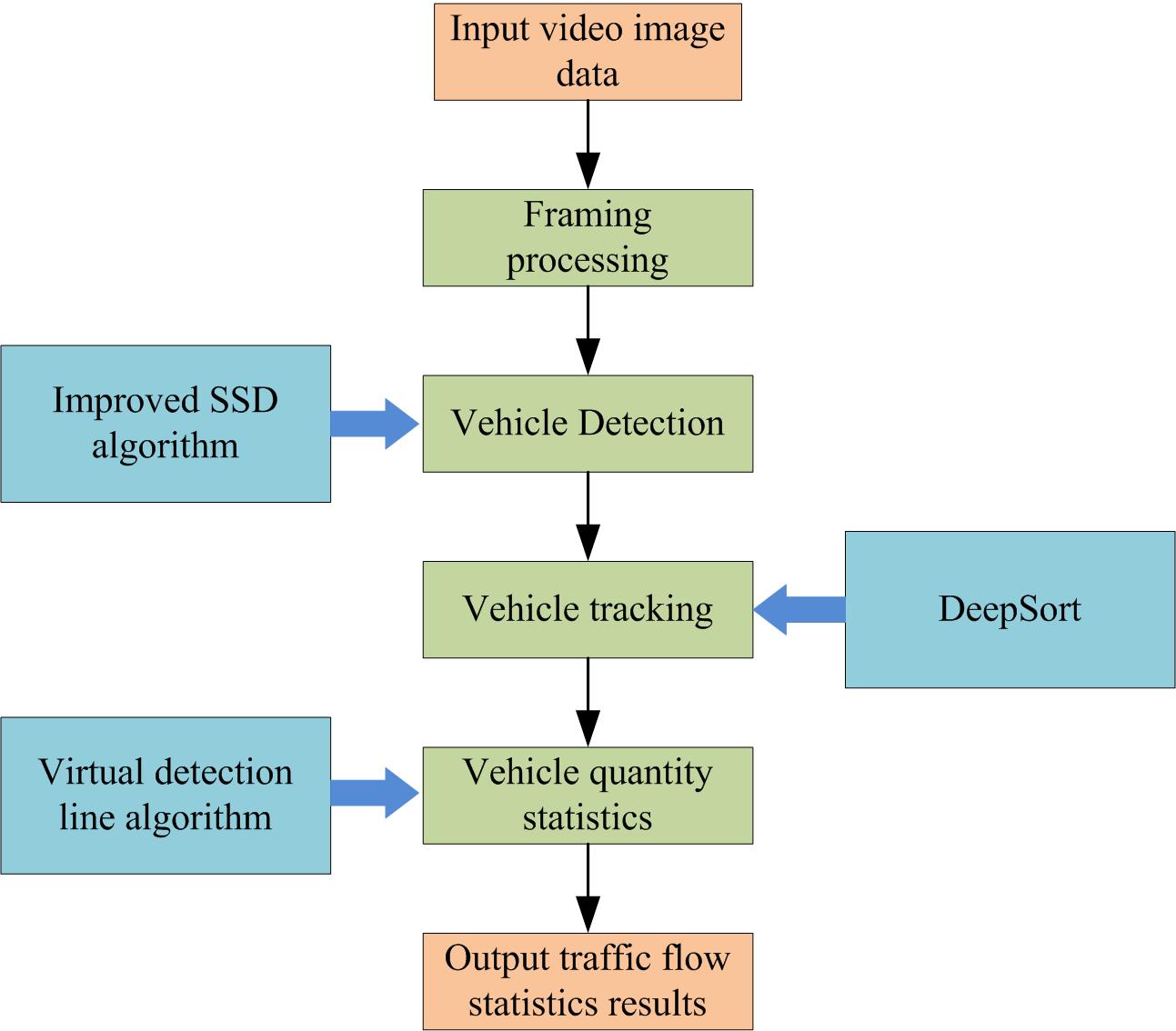

Supplement: S1 File — (ZIP) [file pone.0300214.s001.zip › Supporting information/Figure 6.jpg]

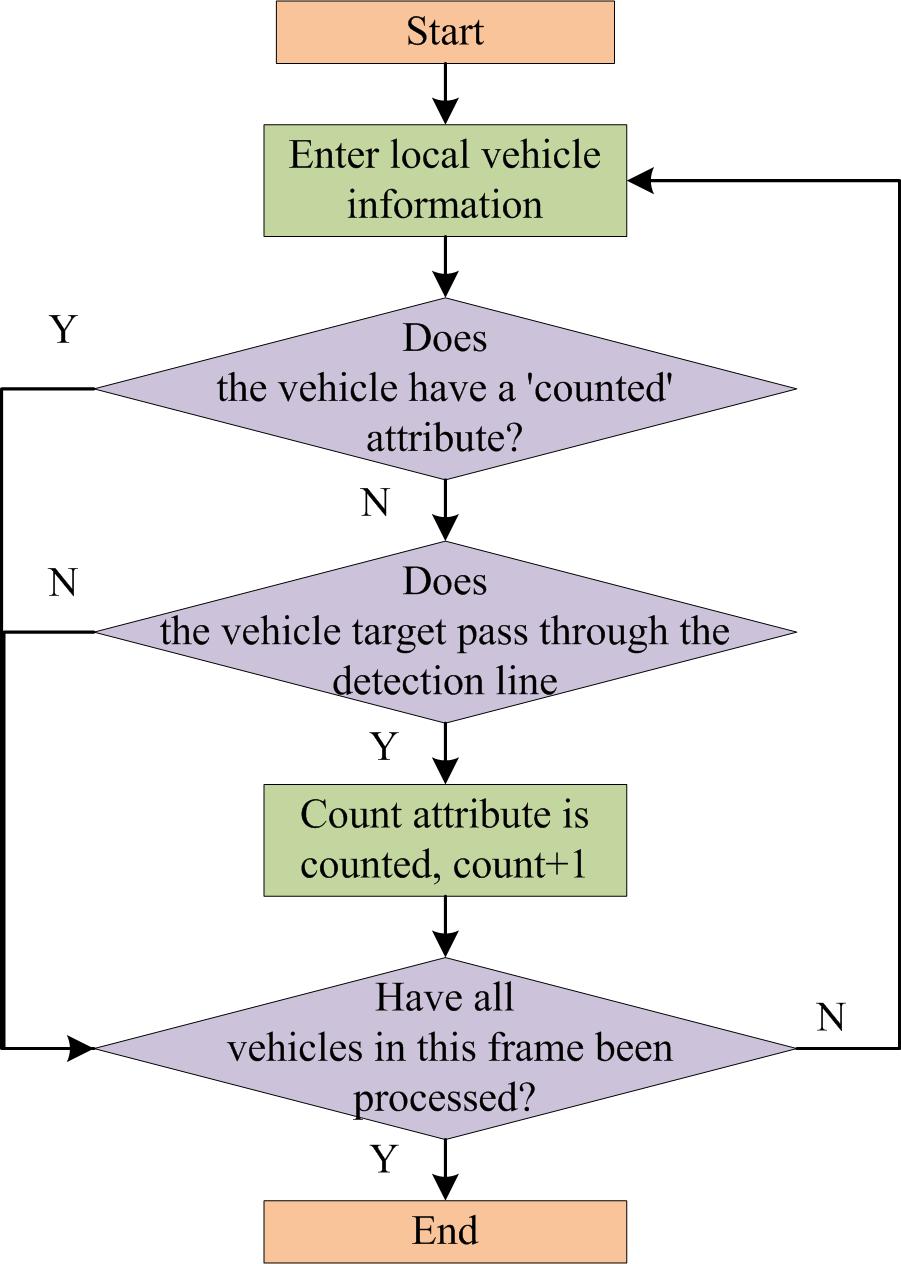

Supplement: S1 File — (ZIP) [file pone.0300214.s001.zip › Supporting information/Figure 7.jpg]

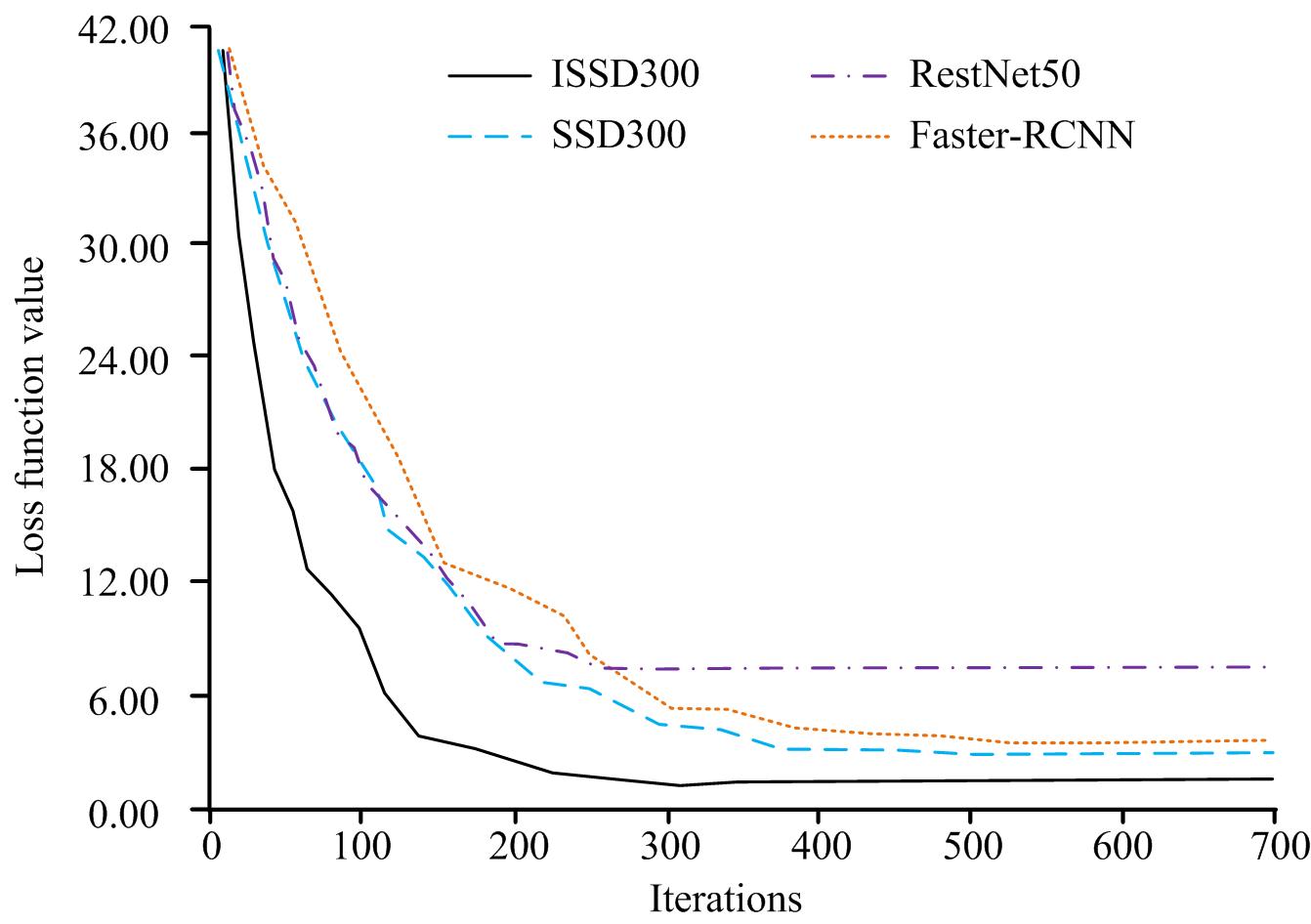

Supplement: S1 File — (ZIP) [file pone.0300214.s001.zip › Supporting information/Figure 8.jpg]

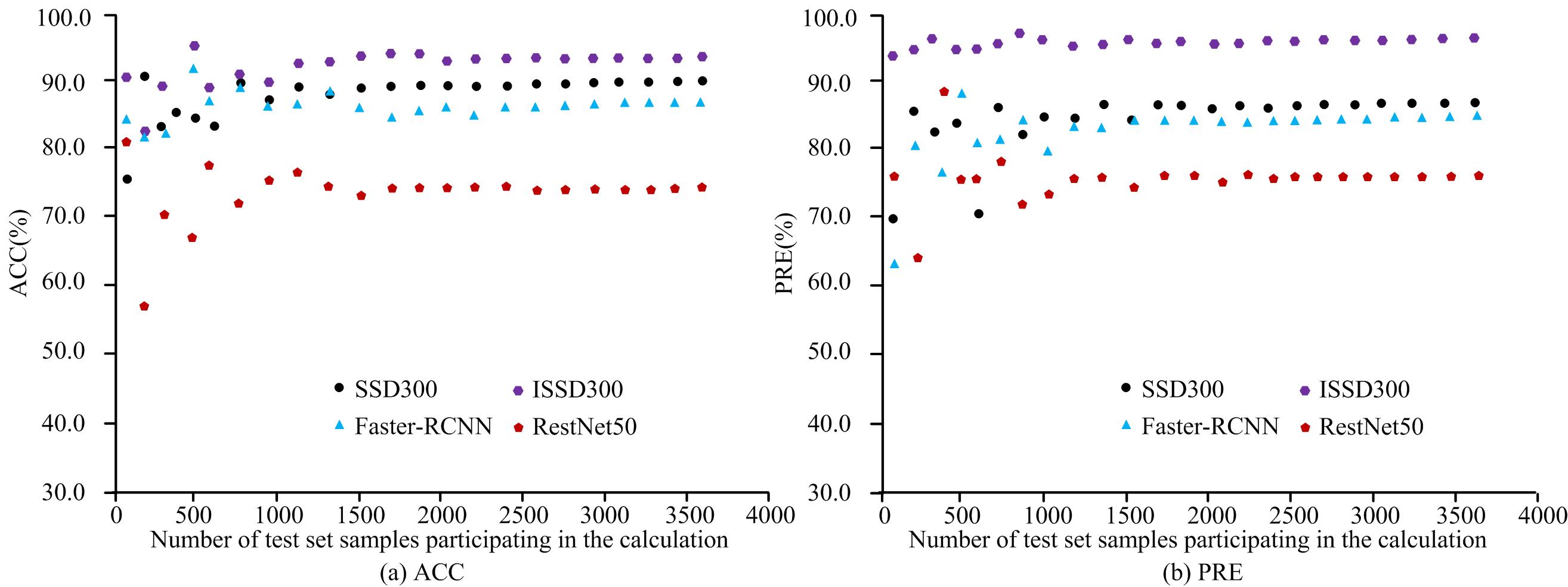

Supplement: S1 File — (ZIP) [file pone.0300214.s001.zip › Supporting information/Figure 9.jpg]
